# *Ustilago maydis* effector Jsi1 interacts with Topless corepressor, hijacking plant jasmonate/ethylene signaling

**DOI:** 10.1111/nph.17116

**Published:** 2021-01-03

**Authors:** Martin Darino, Khong‐Sam Chia, Joana Marques, David Aleksza, Luz Mayela Soto‐Jiménez, Indira Saado, Simon Uhse, Michael Borg, Ruben Betz, Janos Bindics, Krzysztof Zienkiewicz, Ivo Feussner, Yohann Petit‐Houdenot, Armin Djamei

**Affiliations:** ^1^ Gregor Mendel Institute of Molecular Plant Biology (GMI) Austrian Academy of Sciences (OEAW) Vienna BioCenter (VBC) Vienna 1030 Austria; ^2^ The Leibniz Institute of Plant Genetics and Crop Plant Research (IPK) OT Gatersleben 06466 Germany; ^3^ University of Natural Resources and Life Sciences (BOKU) Vienna 1180 Austria; ^4^ Institute of Molecular Biotechnology (IMBA) Vienna BioCenter (VBC) Vienna 1030 Austria; ^5^ Service Unit for Metabolomics and Lipidomics Goettingen Center for Molecular Biosciences (GZMB) University of Goettingen Goettingen D‐37077 Germany; ^6^ Department of Plant Biochemistry Albrecht von Haller Institute and Göttingen Center for Molecular Biosciences (GZMB) University of Göttingen Göttingen D‐37077 Germany; ^7^ UMR BIOGER INRA AgroParisTech Université Paris‐Saclay Thiverval‐Grignon 78850 France; ^8^ The Sainsbury Laboratory University of East Anglia Norwich, NR4 7UK UK; ^9^ Present address: Department of Phytopathology Institute of Crop Science and Resource Conservation University of Bonn Bonn 53115 Germany

**Keywords:** EAR motif, ethylene response factor, jasmonate/ethylene (JA/ET) signaling, Jsi1, Topless, *Ustilago maydis*

## Abstract

- *Ustilago maydis* is the causal agent of maize smut disease. During the colonization process, the fungus secretes effector proteins that suppress immune responses and redirect the host metabolism in favor of the pathogen. As effectors play a critical role during plant colonization, their identification and functional characterization are essential to understanding biotrophy and disease.
- Using biochemical, molecular, and transcriptomic techniques, we performed a functional characterization of the *U. maydis* effector Jasmonate/Ethylene signaling inducer 1 (Jsi1).
- Jsi1 interacts with several members of the plant corepressor family Topless/Topless related (TPL/TPR). Jsi1 expression in *Zea mays* and *Arabidopsis thaliana* leads to transcriptional induction of the ethylene response factor (ERF) branch of the jasmonate/ethylene (JA/ET) signaling pathway. In *A. thaliana*, activation of the ERF branch leads to biotrophic susceptibility. Jsi1 likely activates the ERF branch via an EAR (ET‐responsive element binding‐factor‐associated amphiphilic repression) motif, which resembles EAR motifs from plant ERF transcription factors, that interacts with TPL/TPR proteins.
- EAR‐motif‐containing effector candidates were identified from different fungal species, including *Magnaporthe oryzae*, *Sporisorium scitamineum*, and *Sporisorium reilianum*. Interaction between plant TPL proteins and these effector candidates from biotrophic and hemibiotrophic fungi indicates the convergent evolution of effectors modulating the TPL/TPR corepressor hub.

## Introduction

The biotrophic fungus *Ustilago maydis* causes smut disease on maize (*Zea mays*). During colonization, the fungus secretes manipulative molecules, termed effectors, that interfere with the host’s cellular machinery to suppress plant defense responses, redirect development, and enhance nutrient access (Win *et al*., 2012). As effectors play a critical role during plant colonization, their identification and functional characterization are essential to understanding the process of plant–pathogen interaction. In *U. maydis*, 467 genes were classified as putative secreted proteins (Lanver *et al*., 2017). To date, only a few have been characterized as effector proteins, and they have diverse functions during the biotrophic phase (Djamei *et al*., 2011; Redkar *et al*., 2015; Ma *et al*., 2018).

Plants coordinate pathogen‐specific immune responses through an elaborate crosstalk between hormone signaling pathways. Activation of salicylic acid (SA) signaling usually leads to activation of immune responses against biotrophic and hemibiotrophic pathogens. By contrast, jasmonate (JA) signaling leads to activation of immune responses to necrotrophic pathogens. Whereas ethylene (ET) signaling can be synergistic with JA signaling, SA and JA signaling are generally antagonistic to one another (Pieterse *et al*., 2012). In *Arabidopsis thaliana*, two major branches of the JA signaling pathway have been described. The MYC branch, controlled by MYC‐type transcription factors (TFs), is associated with wound response and defense against herbivorous insects. The ET response factor (ERF) branch is associated with resistance to necrotrophic pathogens. This branch is regulated by members of the APETALA2/ETHYLENE RESPONSE FACTOR (AP2/ERF) family of TFs, like ERF1 and OCTADECANOID‐RESPONSIVE ARABIDOPSIS59 (ORA59), and leads to the transcriptional upregulation of *PLANT DEFENSIN1.2* (*PDF1.2*), a well‐known marker of JA/ET signaling. The ERF branch is co‐regulated by JA and ET signaling (Lorenzo *et al*., 2003; McGrath *et al*., 2005; Dombrecht *et al*., 2007; Pré *et al*., 2008). Some evidence of SA–JA antagonism was shown in monocots, where overexpression of the key SA regulator NONEXPRESSOR OF PATHOGENESIS‐RELATED GENES1 (OsNPR1) is followed by strong induction of SA‐responsive genes and suppression of JA‐responsive genes (Yuan *et al*., 2007). On the other hand, a dichotomy in resistance against biotrophic and necrotrophic pathogens is not quite so simple, as previous studies demonstrated that ET signaling could suppress *Cochliobolus miyabeanus* infection in *A. thaliana* but promote it in *Oryza sativa* (Völz *et al*., 2020).

Pathogens evolved strategies to manipulate defense hormone signaling to render plants more susceptible to infection. Effector proteins from *Pseudomonas syringae* interfere with activity of repressors of the JA signaling, leading to transcriptional activation of JA responses and, thus, promoting bacterial proliferation (Gimenez‐Ibanez *et al*., 2014; Yang *et al*., 2017). How fungal pathogens manipulate JA signaling is only poorly understood. The hemibiotrophic fungal pathogens *Fusarium oxysporum* f.sp. *conglutinans* and *F. oxysporum* f.sp. *matthioli* produce different JA conjugates and exhibit reduced virulence in the *coronatine insensitive1* (*coi1*) mutant, indicating that JA signaling is involved in promoting *Fusarium* infection (Cole *et al*., 2014). Furthermore, previous studies identified JA signaling as a target for both a mutualistic fungus (Plett *et al*., 2014) and a pathogenic fungus (Patkar *et al*., 2015).

The *A. thaliana* TPL/TPR corepressor family is involved in several plant processes, including JA and auxin signaling (Szemenyei *et al*., 2008; Pauwels *et al*., 2010) and defense responses (Zhu *et al*., 2010). TPL/TPR proteins contain several conserved domains. The N‐terminal portion contains LIS1 homology (LisH), C‐terminal to LisH (CTLH), and CT11‐RanBPM (CRA) domains. The C‐terminal portion contains two WD40 domains (Martin‐Arevalillo *et al*., 2017). TPL/TPRs can interact with transcriptional regulators via short repression domains (Causier *et al*., 2012b) which include the ethylene‐responsive element binding‐factor‐associated amphiphilic repression (EAR) motif, defined by a consensus sequence of either LxLxL or DLNxxP (Kagale *et al*., 2010). Proteins with an LxLxL motif have been found to interact with the N‐terminal portion of TPL/TPR proteins (Szemenyei *et al*., 2008; Pauwels *et al*., 2010). By contrast, proteins with a DLNxxP motif interact with the C‐terminal portion of TPL/TPR proteins (Liu *et al*., 2019), but it is not known which of the two WD40 domains is responsible for the interaction with the DLNxxP motif, and the contribution of the C‐terminal portion to the transcriptional repression activity of the TPL/TPR proteins is also unclear. XopD, an effector possessing two LxLxL EAR motifs, was identified in the plant pathogen *Xanthomonas euvesicatoria* (Kim *et al*., 2013). XopD binds *SlERF4*, and its EAR motif is required for suppression of the plant immune response. Additionally, an effector from *Ralstonia solanacearum*, PopP2, possesses an LxLxL EAR motif that is required for avirulence and protein stability (Segonzac *et al*., 2017). No EAR‐motif‐containing effectors have been reported in any *Ustilago* pathosystem so far.

Here, we demonstrate that the *U. maydis* effector Jsi1 possesses a DLNxxP motif that interacts with the second WD40 domain of TPL/TPRs. Upon expression in *A. thaliana*, Jsi1 leads to induction of genes related to the ERF branch of JA/ET signaling, suggesting that binding to the second WD40 domain of TPL/TPRs may trigger this branch of the JA/ET signaling pathway. In addition, *A. thaliana* plants expressing Jsi1 are more susceptible to *P. syringae* infection, which would correlate with the induction of the ERF branch. In maize, Jsi1‐dependent interaction with TPL/TPRs leads to induction of ERF genes that could be associated with ERF‐branch activation in maize. Jsi1 could activate the ERF branch by interfering with the activity of endogenous DLNxxP‐motif‐containing ERF TFs. The identification of unrelated effector proteins from different fungal species with a DLNxxP motif and validation of the interaction between *Magnaporthe oryzae*, *Sporisorium scitamineum*, and *Sporisorium reilianum* effectors with TPL/TPRs indicate the convergent evolution of a strategy to manipulate this signaling hub in plants.

## Material and Methods

### Plant material, growth conditions, and plasmids

*Zea mays* cv Early Golden Bantam (EGB; Olds Seeds, Madison, WI, USA) was used for infection with *U. maydis*. Maize were grown in a glasshouse (16 h : 8 h, light : dark cycle, 28°C : 20°C). *Nicotiana benthamiana* plants were grown in a growth chamber (16 h : 8 h, light : dark cycle, 22°C, 60% humidity). *Arabidopsis thaliana* β‐estradiol inducible lines XVE‐jsi1‐mCherry and control XVE‐mCherry lines were created by transfer DNA insertion in Col‐0 background. *Arabidopsis thaliana* plants were grown in a growth chamber (12 h : 12 h, light : dark cycle, 21°C, 60% humidity). All plasmids used in this work are provided in Supporting Information Table S1. Detailed cloning, gene accession numbers, virulence assay and phytohormone measurements are provided in Methods S1.

### Secretion experiments in axenic culture and *in planta*

*Ustilago maydis* strain AB33P_otef_ *jsi1‐3xHA* was generated through insertion of plasmid pUG‐P_otef_‐*Jsi1*‐3xHA into the *ip* locus of AB33 according to Aichinger *et al*. (2003). We performed the secretion assay according to Brachmann *et al*. (2001). Mouse monoclonal anti‐hemagglutinin (HA; Sigma Aldrich) and anti‐actin (Invitrogen) antibodies were used for Western blot. The experiment was repeated with three independent transformant strains with similar results.

To visualize protein secretion *in planta*, we generated the SG200Δ*jsi1*P_cmu1_ *Jsi1mCherry* strain by integrating *Jsi1‐mCherry* under control of the *cmu1* promoter in the *ip* locus. In addition, we built a nonsecreted version of the Jsi1‐mCherry strain (SG200P_cmu1_ *Jsi1_27641_mCherry*). We independently infected both strains in 7‐d‐old maize seedlings. mCherry fluorescence signal was detected using confocal microscopy at 3 d postinfection (dpi).

### Yeast two‐hybrid assay

We performed yeast two‐hybrid (Y2H) assays with the Matchmaker™ GAL4 Two hybrid system (Clontech®, Mountain View, CA, USA) following the manufacturer's protocol. We fused the GAL4 activation domain of the prey vector pGG446 (modified version of pGADT7) to the genes *Jsi1_27641_*, *Jsi1m_27641_*_,_ *ZmERF4*, and yellow fluorescent protein (*YFP*). We fused the GAL4 binding domain from the bait vector pGG187 (modified version of pGBKT7) to the genes *ZmTPL1*, *ZmTPL2*, *ZmTPL3*, *TPL* (*AT1G15750*), *TPR1* (*AT1G80490*), *TPR2* (*AT3G16830*), *TPR4* (AT3G15880), *YFP*, and N and C‐terminal portions of the different topless orthologues. We transformed the combinations of pGG446 and pGG187 vectors carrying the different genes in the yeast strains Y187 (MAT α) and AH109 (MAT a), respectively. We selected diploid yeast after mating for growth on (SD)−Leu/−Trp and (SD)−Leu/−Trp/−His plates at 28°C for 4 d. We repeated the experiments twice from independent mating events.

### Co‐immunoprecipitation assay in *N. benthamiana* and *Z. mays*

We infiltrated 4‐wk‐old *N. benthamiana* leaves with *Agrobacterium tumefaciens* carrying different genes cloned into an expression vector as described (Ma *et al*., 2012). Cultures carrying the different gene combinations were infiltrated in six leaves (three plants, two leaves from each plant). A 450 mg sample of tissue powder was suspended in 2 ml cold extraction buffer for protein extraction (50 mM Hepes pH 7.5, 100 mM sodium chloride, 10% v/v glycerol, 1 mM EDTA, 0.1% v/v Triton X‐100, 2% polyvinylpolypyrrolidone, 1 mM dithiothreitol, 1 mM phenylmethanesulfonylfluoride, and EDTA‐Free Protease Inhibitor cocktail; Roche). Protein pull‐down was performed using the μMACS™ MicroBeads system from Miltenyi Biotech (Bergisch Gladbach, Germany) following the manufacturer's instructions.

We quantified protein signals of ZmTPL1, ZmERF4, and the different versions of Jsi1 in the input. The protein signals of ZmTPL1, ZmERF4, and the different versions of Jsi1 were normalized to the respective Rubisco (Ponceau) protein signal. Fold change (FC) for each protein was shown relative to the normalized protein value observed in ZmTPL1 co‐expressed with YFP or Jsi1 and ZmERF4. Quantification of pulled‐down proteins signal of ZmERF4 and the different versions of Jsi1 were normalized to their respective pulled‐down ZmTPL1 protein signal. FC for each protein was shown relative to the value in ZmTPL1 co‐expressed with Jsi1 and ZmERF4. FC ± SD values are means of three biological replicates for all the experiments.

In the case of maize, *U. maydis* strains SG200P_cmu1_ *jsi1‐3xHA*, SG200P_cmu1_ *jsi1m‐3xHA*, and SG200P_cmu1_‐SP_cmu1(1‐22)_‐*mCherry‐3xHA* were generated by integration of the different constructs into the *ip* locus of SG200. We infected 7‐d‐old seedlings with each strain (30 plants per strains). Infected tissue was collected 7 dpi. The co‐immunoprecipitation (Co‐IP) protocol was the same as for *N. benthamiana*.

We detected the immunoprecipitated proteins with anti‐MYC (Sigma Aldrich), anti‐HA, anti‐mCherry (Abcam), or anti‐green fluorescent protein (GFP; Miltenyi Biotech) antibodies depending on the experiment. The TPL‐specific antibody was raised using a small peptide, CNEQLSKYGDTKSAR, selected from a conserved region of the TPL/TPR proteins. The polyclonal antibody was produced in rabbit by Eurogentec (Seraing, Belgium). We repeated each experiment three times.

### *Arabidopsis thaliana* RNA‐sequencing sample collection

*Arabidopsis thaliana* seeds from XVE‐jsi1‐mCherry‐1/2 and XVE‐mCherry lines were grown vertically on square plates containing Murashige & Skoog medium for 7 d. *Arabidopsis thaliana* seedlings were transferred to square plates with the same media containing 5 µM β‐estradiol and incubated for 6 h. Three independent replicates for each genotype were collected. Mock treatment was only performed for the control line to confirm that the concentration of β‐estradiol used for the experiment did not itself alter gene‐expression.

### RNA‐sequencing analysis

We removed adapter sequences and performed quality trimming using Trimmomatic (Bolger *et al*., 2014). Reads were mapped to the reference genome using Star, v.2.7.0e (Dobin *et al*., 2013) with the parameter outFilterMismatchNoverLmax 0.05. We input the bam files to R v.3.5.1 using the package R/samtools. We obtained the genome annotation from Araport11 and gene models and read counts per gene were obtained with the packages Genomic Features and Genomic Alignments, respectively. We removed low‐expressed genes and analyzed 28 843 for differential expression using DESeq2 after performing regularized log transformation (Love *et al*., 2014). We compared all the replicates from β‐estradiol‐induced XVE‐jsi1‐mCherry lines with the replicates from control XVE‐mCherry lines with and without β‐estradiol induction and kept genes with log FC ˃ 1.5 and adjusted *P* < 0.05. We performed Gene Ontology (GO)‐term analysis for biological processes using the ThaleMine tool (Krishnakumar *et al*., 2014). The data sets were deposited in National Center for Biotechnology Information’s (NCBI's) Gene Expression Omnibus and are accessible through GEO Series accession no. GSE142128 (https://www.ncbi.nlm.nih.gov/geo/query/acc.cgi?acc=GSE142128).

To assess the significance of enrichment for TF binding sites, we first determined the direct target genes of *ERF3*, *4*, *7*, *8*, *10*, *11* and *ZAT10* using available DNA affinity purification sequencing (DAP‐seq) data (O’Malley *et al*., 2016). We overlapped each list of putative direct target genes with genes upregulated upon *jsi1* induction (FC ˃ 1.5, *P* < 0.05). We determined the significance of the overlapping genes with Fisher's exact test using the R package geneoverlap function newGOM (https://github.com/shenlab‐sinai/GeneOverlap).

### Reverse transcription PCR for RNA‐sequencing validation

Total RNA was extracted from three independent replicates from each *A. thaliana* line (XVE‐jsi1‐mCherry‐1 and 2 and XVE‐mCherry) using the same protocol for RNA‐sequencing (RNA‐seq) samples. Complemetary DNA (cDNA) was generated from total RNA using the iScript cDNA synthesis kit (Bio‐Rad). We performed quantitative reverse transcription (qRT)‐PCR using FastStart Universal SYBR Green Master mix (Roche) according to the manufacturer's instructions. The relative amount of amplicons in the samples were calculated with the $2^{‐\Delta \Delta C_{t}}$ method (Livak & Schmittgen, 2001) with *actin2* (*AT3G18780*) as the reference gene (Czechowski *et al*., 2005). We calculated FC in the expression level of each gene in the XVE‐jsi1‐mCherry lines compared with the XVE‐mCherry line, and data are represented for each jsi1‐mCherry line as the mean of three replicates. We calculated statistically significant differences in gene expression between each jsi1‐mCherry line and mCherry line using ANOVA followed by Dunnett’s multiple comparison test with *P* < 0.05.

For evaluation of the induction of the ERF branch in maize, 10 EGB seedlings infected with SG200 were collected at 4 and 6 dpi. Mock seedlings were infected with water, and tissue was collected for each time point. Three independent replicates were performed for infected and mock tissue at each time point. RNA extraction, cDNA synthesis, and qRT‐PCR and data analysis were performed as previously described but using a cyclin‐dependent kinase (CDK; GRMZM2G149286) as the reference gene (Lin *et al*., 2014). Primers used for RT‐PCR are described in Table S2.

### Biolistic transformation of maize for localization and gene induction analysis

We bombarded 6‐d‐old maize leaves with 1.6 µm gold particles coated with 5 µg of each plasmid as described by Djamei *et al.,* (2011). Fluorescence emission was observed 1 d after transformation by confocal microscopy. For gene induction analysis, we bombarded 7 µg of the corresponding plasmids (35S‐Jsi1‐mcherry or 35S‐Jsi1m‐mcherry) into 12‐d‐old maize leaves. Samples were harvested 10 h after bombardment for RNA extraction and qRT‐PCR.

### Identification of putative secreted effector proteins with a DLNxxP motif

We downloaded predicted protein sequences of the different plant pathogens from EnsemblFungi (https://fungi.ensembl.org/index.html) or NCBI (https://www.ncbi.nlm.nih.gov/). To identify putative secreted effector proteins with a DLNxxP motif, we searched for the DLNxxP motif in all predicted proteins from the different fungal species using CLC Main Workbench 7.7.2 (Qiagen). Among all the DLNxxP‐motif‐containing proteins, we searched for those with a predicted secretion signal (SignalIP‐5.0), lacking transmembrane domains (Tmhmm v.2.0 from http://www.cbs.dtu.dk/services/), and no predicted enzymatic domains (InterPro, https://www.ebi.ac.uk/interpro/beta/).

## Results

### Jsi1 interacts with the C‐terminal portion of Topless

**Fig. 1:**
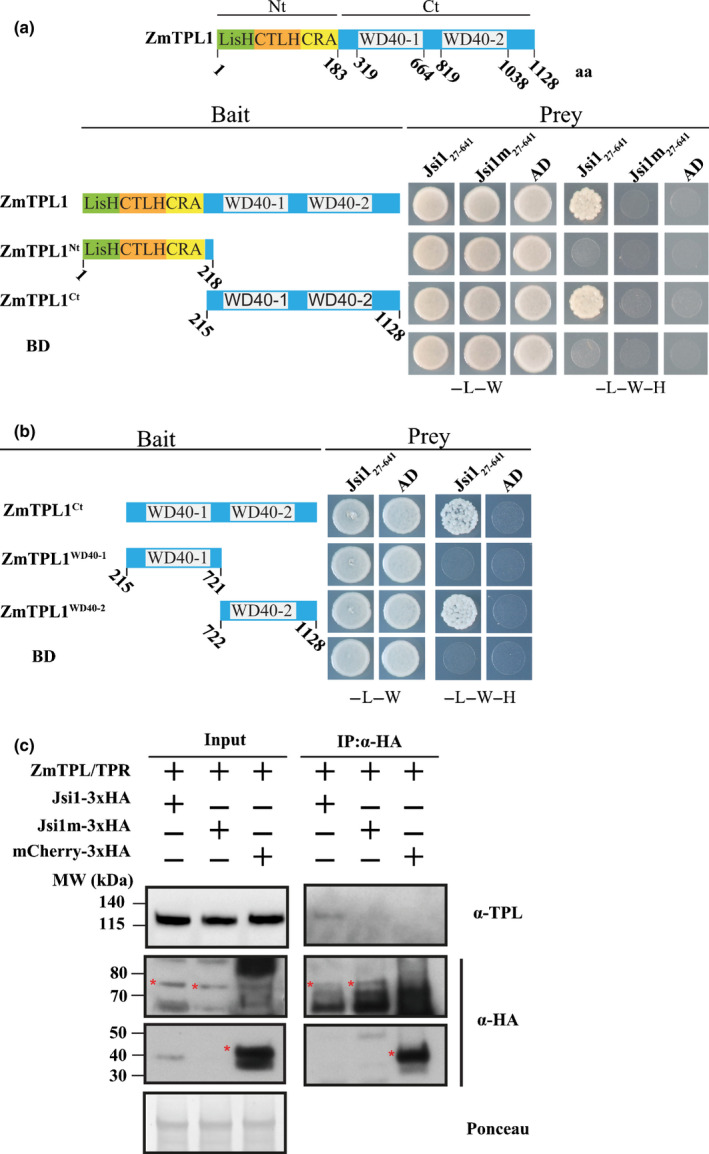
Jasmonate/Ethylene signaling inducer 1 (Jsi1) interacts with the second WD40 domain of ZmTPL1 through its DLNxxP motif. (a) Yeast two‐hybrid (Y2H) assay with Jsi1_27641_ or the mutant version Jsi1m_27641_ as prey and full‐length ZmTPL1 or its N and C‐terminal regions (ZmTPL1^Nt^ and ZmTPL1^Ct^) as bait. (b) ZmTPL1^WD401^ and ZmTPL1^WD402^ each containing one of the WD40 repeats were used as baits to test which WD40 domain interacts with Jsi1 in Y2H assay. As a negative control, we used enhanced yellow fluorescent protein fused to the GAL4‐binding domain (BD) and GAL4 activation domain (AD). −L, −W and −H indicate medium lacking leucine, tryptophan, and histidine, respectively. (c) Co‐immunoprecipitation (Co‐IP) assay showing that Jsi1 interacts with ZmTPL/TPRs in *Zea mays*. We infected maize seedlings with *Ustilago maydis* strains expressing Jsi1‐3xHA, Jsi1m‐3xHA and mCherry‐3xHA and performed a Co‐IP using anti‐hemagglutinin (HA) antibody. Topless (TPL)‐specific antibody shows that endogenous maize Topless/Topless related (TPL/TPR) proteins are co‐purified with Jsi1‐3xHA but not with Jsi1m‐3xHA or mCherry‐3xHA. Red asterisks indicate the full‐length proteins of Jsi1‐3xHA, Jsi1m‐3xHA and mCherry‐3HA. Ponceau staining was used to ensure equal loading. To detect mCherry, membranes were exposed between 15 and 30 min, whereas for Jsi1 and Jsi1m the membranes were exposed longer, between 3 and 4 h.

As EAR‐motif‐containing effectors can be important for the establishment of plant‐pathogen interactions, we screened putative *U. maydis* effector proteins for the presence of the DLNxxP EAR motif and identified the gene *jsi1* (*UMAG_01236),* located in the effector cluster 2A (Table 1; Fig. S1a; Kämper *et al*., 2006). *Jsi1* is transcriptionally induced during biotrophy and its expression peaks 4 dpi (Fig. S1b). To test if Jsi1 without signal peptide (Jsi1_27641_) can interact with TPL, we cloned three *Z. mays* TPL orthologues: *ZmTpl1*, Z*mTpl2* and *ZmTpl3* (Fig. S1c). Jsi1_27641_ interacts with all three ZmTPLs in Y2H assays (Figs 1a, S1d). To identify which TPL domain interacts with Jsi1, we split ZmTPL1 into its N‐terminal portion comprising the LisH, CTLH, and CRA domains (ZmTPL1^Nt^) and C‐terminal portion containing the WD40 domains (ZmTPL^Ct^). We found that Jsi1_27641_ interacts with ZmTPL^Ct^ in Y2H assays (Fig. 1a). To identify which of the two WD40 repeats is responsible for this interaction, we further divided ZmTPL^Ct^ into two fragments, each containing a single WD40 repeat (WD40‐1 and WD40‐2) and tested them for interaction by Y2H assays. Jsi1_27641_ specifically interacted with WD40‐2 (Fig. 1b). To determine if Jsi1 is able to interact with ZmTPL/TPR proteins in maize, we created a *U. maydis* strain expressing Jsi1‐3xHA under the control of the strong biotrophy‐induced *cmu1* promoter to increase the protein expression level of Jsi1 during infection. In addition, we raised an anti‐TPL antibody that was tested for specificity with TPL proteins from different plant species (Fig. S1e). We immunoprecipitated Jsi1‐3xHA from infected maize seedlings and were able to detect co‐immunoprecipitated TPL/TPR proteins by Western blot (Figs 1c, S2). To test the specificity of the Jsi1/TPL interaction, we mutated the DLNxxP EAR motif in Jsi1 to AHNxxP (Jsi1m). We found that Jsi1m did not interact with ZmTPL1 in either Y2H or *in planta* Co‐IP assays (Fig. 1a,c), indicating a critical role for the DLNxxP EAR motif in the interaction between Jsi1 and TPL/TPR proteins.

**Table 1:** Fungal effector proteins possessing an DLNxxP motif.

Protein ID	Length (aa)	SignalP^a^		DLNxxP		Species	Lifestyle
		Score signal	Cleavage site (aa)	Location (aa)	Sequence motif		
UMAG_01236	641	0.8706	26	39–44	DLNELP	*Ustilago maydis*	Biotrophic
UMAG_01237	633	0.9817	21	36–41	DLNKLP		
UMAG_05303	193	0.991	21	53–58	DLNFHP		
UMAG_02826	399	0.9677	22	251–256	DLNIAP		
Sr10432	120	0.9884	23	104–109	DLNKHP	*Sporisorium reilianum*	Biotrophic
Sr10312	631	0.9564	23	36–41	DLNEIP		
Sr13382	289	0.9961	21	48–53	DLNQPP		
SPSC_03537	653	0.9437	20	27–32	DLNKIP	*Sporisorium scitamineum*	Biotrophic
PTTG_28402	394	0.9663	31	57–62	DLNSIP	*Puccinia triticina*	Biotrophic
PTTG_07660	442	0.9345	24	135–140	DLNGTP		
PTTG_27442	229	0.9321	23	38–43	DLNEFP		
PTTG_27452	529	0.9315	23	38–43	DLNEFP		
PTTG_27005	407	0.921	25	30–35	DLNLPP		
PTTG_26956	418	0.728	25	312–317	DLNDRP		
PTTG_05870	213	0.8818	21	77–82	DLNNVP		
PTTG_26367	313	0.6855	19	40–45	DLNEYP		
PTTG_25346	473	0.7534	23	37–42	DLNAFP		
CSEP0438	227	0.9855	26	145–150	DLNYYP	*Blumeria graminis*	Biotrophic
FOXG_20822	81	0.993	17	23–28	DLNRDP	*Fusarium oxysporum*	Hemibiotrophic
MGG_15391	222	0.8834	23	94–99	DLNKAP	*Magnaporthe oryzae*	Hemibiotrophic
MGG_05887	247	0.9944	16	131–136	DLNKVP		

a Program used to bioinformatically predict the secretion signal in proteins (http://www.cbs.dtu.dk/services/signalP/). aa, amino acids.

### Jsi1 is a secreted effector located in the nucleus of maize cell leaves

**Fig. 2:**
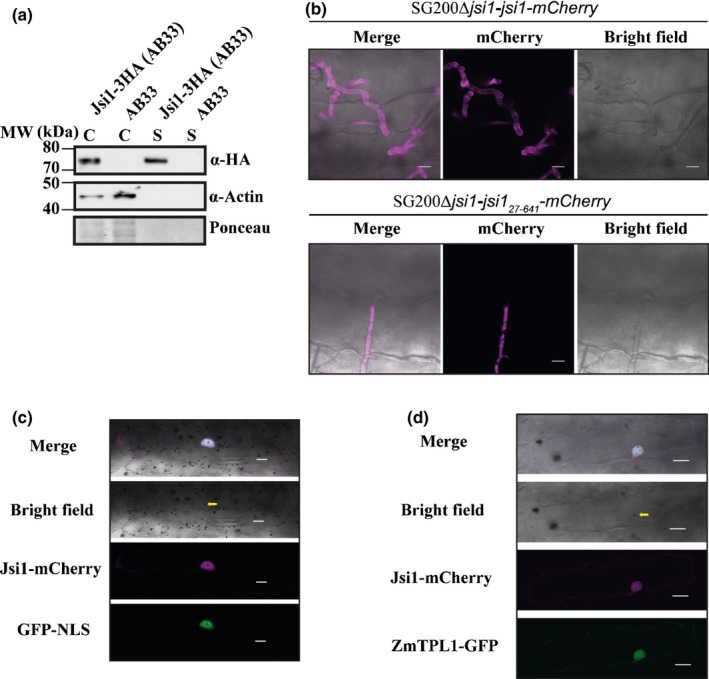
Jasmonate/Ethylene signaling inducer 1 (Jsi1) is a secreted effector that is targeted to the plant cell nucleus. (a) Jsi1 is secreted in axenic culture. We expressed Jsi1‐3xHA in the *Ustilago maydis* strain AB33. Proteins from filamentous cells and culture supernatants were subjected to Western blot analysis using anti‐hemagglutinin (HA) and anti‐actin antibodies. We used actin as a control of cell lysis, and it was only detected in whole‐cell extracts (C). Jsi1‐3xHA was detected in whole cell extracts (C) and culture supernatants (S). (b) Jsi1‐mCherry is secreted by *U. maydis* in maize. Confocal images of infected maize leaves 3 d postinfection with SG200*Δjsi1*‐*Jsi1*‐*mCherry* and SG200*Δjsi1*‐*Jsi1_27641_*‐*mCherry* (a nonsecreted version of Jsi1). Bars, 10 µm. (c) Jsi1 localizes to the nucleus of maize cells. Maize cells expressing Jsi1_27641_‐mCherry and green fluorescent protein–nuclear localization signal (GFP‐NLS) as a nuclear marker after biolistic transformation of leaves. (d) Jsi1 and ZmTPL1 co‐localize in the nucleus of maize leaf cells. Maize cells expressing Jsi1_27641_‐mCherry and ZmTPL1‐GFP. The yellow arrow indicates the transformed cell with the gold particle inside the nucleus. Bars, 20 µm.

To test whether Jsi1 is secreted, we integrated a version of Jsi1‐3xHA into *U. maydis* strain AB33, which is commonly used to study effector secretion (Tollot *et al*., 2016). As expected for a secreted protein, we detect Jsi1‐3xHA in the culture supernatant by Western blot. Actin, which served as a lysis control, was only present in whole‐cell extracts (Fig. 2a). To confirm that Jsi1 is secreted *in planta*, we expressed a Jsi1‐mCherry fusion protein in strain SG200Δ*jsi1*, where the endogenous *jsi1* locus was deleted. To increase protein levels for visualization, *jsi1‐mCherry* was expressed by the *cmu1* promoter. Jsi1_27641_‐mCherry, without signal peptide, was used as a negative control. We observed Jsi1‐mCherry fluorescence around and outside the fungal hyphae whereas Jsi1_27641_‐mCherry was localized inside the fungal hyphae, indicating that Jsi1 is secreted by *U. maydis in planta* (Fig. 2b). To determine the subcellular localization of Jsi1 in maize cells, we transiently co‐transformed a Jsi1_27641_‐mCherry construct with a GFP‐nuclear localization signal construct as a nuclear marker into maize leaves. Using confocal microscopy, we found Jsi1_27641_‐mCherry signal inside the plant nucleus 1 d after biolistic transformation (Fig. 2c). To test whether ZmTPL1 colocalizes with Jsi1 in maize cells, we co‐transformed a ZmTPL1‐GFP construct with Jsi1_27641_‐mCherry. ZmTPL1‐GFP signal emission overlapped with the Jsi1_27641_‐mCherry signal in the nucleus, indicating co‐localization of both proteins (Fig. 2d).

*Jsi1* belongs to the *U. maydis* cluster 2a, which was previously shown to cause a mild hypervirulence phenotype in maize when deleted (Kämper *et al*., 2006). To test whether Jsi1 contributes to virulence, we infected maize seedlings with three independent strains mutated in the *jsi1* locus (SG200Δ*jsi1* 1 to 3). SG200Δ*jsi1* mutant strains showed no impaired ability to filament on charcoal, a prerequisite for infection (Fig. S1f). Plants infected with the mutant strains did not show any significant changes in symptom development 12 dpi when compared with plants infected with SG200 (Fig. S1g), which may indicate redundancy with other effectors possessing an EAR motif (Table 1).

### Jsi1 activates jasmonate/ethylene and salicylic acid signaling in *A. thaliana*

Since Jsi1_27641_ also binds to TPL/TPR proteins from *A. thaliana* (Fig. S3a), we were able to study which pathways are manipulated by Jsi1 *in planta*. We generated two independent *A. thaliana* lines expressing Jsi1_27‐641_‐mCherry under the control of the estradiol‐inducible XVE system (XVE‐jsi1‐mCh 1 and 2) as well as an XVE‐mCherry (XVE‐mCh) control line. We confirmed expression of the transgenes 6 h after β‐estradiol induction by Western blotting (Fig. S3b) and then subjected induced samples to RNA‐seq. Principal component analysis of the resulting transcriptomes showed that replicates from the two Jsi1 lines group together and are separate from the replicates of the control line, which also clustered (Fig. S3c). Using cutoffs of FC > 1.5 and *P* < 0.05, we identified 1090 differentially expressed genes (DEGs) in Jsi1 lines relative to the control, 915 of which were upregulated and 175 were downregulated. The more than five times higher number of upregulated than downregulated genes is consistent with the model that Jsi1 interferes with the repressor‐function of TPL/TPR proteins. GO‐term analysis for biological processes show several categories related to ‘responses to different stimulus’. Within these, ‘responses to stress’, ‘defense responses’, ‘responses to external stimulus’, and ‘response to biotic stimulus’ were the major categories with 26% to 12% of the total DEGs. This indicates that Jsi1 induces plant immune responses in *A. thaliana* (Fig. S4a). In addition, we identified two GO categories related to hormone responses: ‘response to salicylic acid’ and ‘ethylene response genes’.

**Fig. 3:**
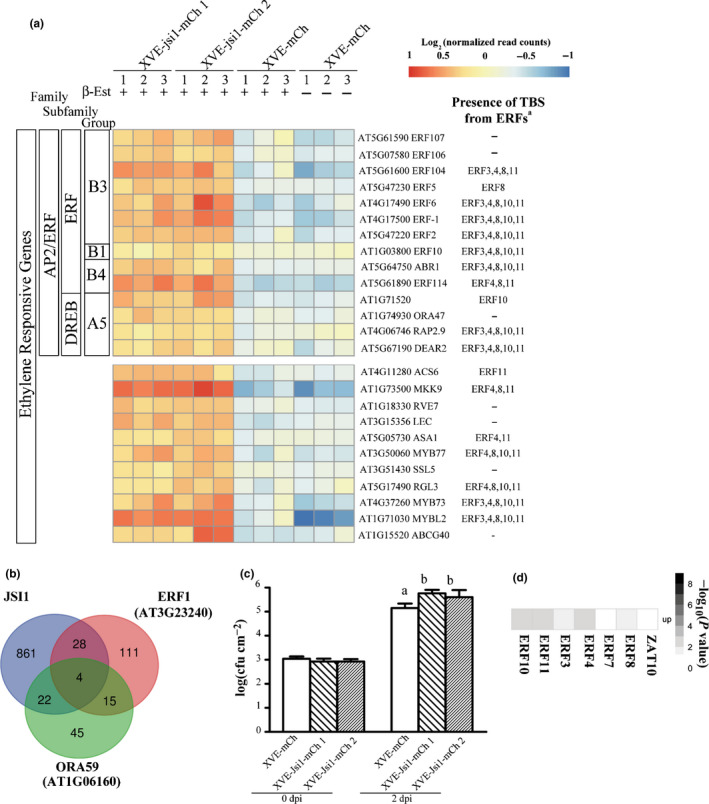
Jasmonate/Ethylene signaling inducer 1 (Jsi1) activates jasmonate/ethylene signaling leading to biotrophic susceptibility. (a) Heat map from RNA sequencing showing ethylene‐responsive genes. Numbers under the lines represent the replicate number. ^†^Genes enriched in transcription binding sites (TBSs) from ethylene response factors (ERFs) with repression activity. (b) Venn diagram showing transcriptionally induced genes shared by *Arabidopsis thaliana* plants expressing either Jsi1, ERF1 or ORA59. (c) *Pseudomonas syringae* pv *tomato* (*Pst*) DC3000 proliferate better in *A. thaliana* plants expressing Jsi1. Infected leaves were collected at 0 d postinfection (dpi) and 2 dpi to quantify bacterial proliferation. The graph shows one representative replicate of three repeated experiments. Different letters indicate statistically significant differences among the different genotypes, which were calculated by Tukey's honestly significant difference post‐hoc test (*P* < 0.05). log(CFU cm^−2^) ± SD: log scale of colony forming units per square centimeter. (d) Genes upregulated upon *jsi1* induction are enriched in TBSs of ERFs with a DLNxxP motif. Matrix summarizing the overlap enrichment between putative direct target genes of ERFs and ZAT10 from previously available DNA affinity purification sequencing data and genes upregulated upon *jsi1* induction. Significance of enrichment of TBSs for each TF was determined by Fisher’s exact test (*P* < 0.05).

In the ET response gene category, 14 DEGs belong to the AP2/ERF family of TFs. Seven of these belong to the B3 group of the ERF subfamily, which are characterized as being positive regulators of transcription. Of these, *ERF2*, *ERF5*, *ERF6* and *ERF107* have been associated with defense responses against necrotrophic infections and are positive regulators of the defense response gene *PDF1.2* (McGrath *et al*., 2005; Moffat *et al*., 2012; Ju *et al*., 2017) (Fig. 3a; Table S3). Two other TFs of the B3 group, *ERF1* and *ORA59*, were found to be transcriptionally controlled by JA and ET and induce *PDF1.2* expression (Lorenzo *et al*., 2003; Pré *et al*., 2008). Even though we did not find *ERF1* and *ORA59* to be upregulated upon *jsi1* induction (Fig. S3e), a comparison of genes upregulated by Jsi1 and those found to be induced by *ERF1* and *ORA59* in previous studies show a 20% and 30% overlap, respectively (Fig. 3b). Among the upregulated genes shared by Jsi1 with ERF1 and/or ORA59, we note the defense‐related genes *OSM34*, *PR5* and *PDF1.2* (Table S3). In addition, Jsi1 also induces three 1‐aminocyclopropane‐1‐carboxylate synthases (ACSs), *ACS2*, *ACS6*, and *ACS11*, and *MAP KINASE KINASE 9* (*MKK9*), which are involved in ET biosynthesis (Xu *et al*., 2008; Tsuchisaka *et al*., 2009) (Table S3). We validated the expression of some *ERF*s, *ACS*s, *MKK9*, and defense‐related genes by qRT‐PCR upon *jsi1* expression (Fig. S3 **e**). Taken together, our results show that Jsi1 induces the expression of several ERFs, genes related with ET synthesis and defense genes, including *PDF1.2*, indicating that Jsi1 induces the ERF branch of the JA/ET signaling pathways.

**Fig. 4:**
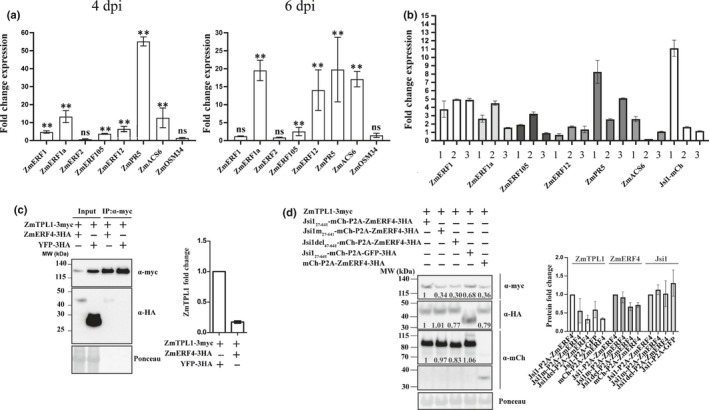
Induction of ethylene response factor (ERF)‐branch genes upon *Ustilago maydis* infection and Jasmonate/Ethylene signaling inducer 1 (Jsi1) overexpression. (a) Quantitative reverse transcription (qRT)‐PCR evaluation of maize orthologues of ERF branch upon *U. maydis* infection at 4 d postinfection (dpi) and 6 dpi. Fold change (FC) ± SD is relative to the expression in maize plants without *U. maydis* infection and normalized to the cyclin‐dependent kinase (CDK) RNA expression values. Values shown are the means of three replicates. Statistically significant differences between genes expressed in *U. maydis*‐infected maize tissue and mock were calculated using Mann–Whitney test (**, *P* < 0.01; ns, not significant). (b) qRT‐PCR evaluation of maize orthologues to the ERF branch upon Jsi1‐mcherry overexpression via biolistic bombardment. Each bar represents an independent biological replicate, three replicates per gene. FC ± SD is relative to the expression observed upon Jsi1m‐mcherry expression and normalized to the CDK RNA expression values. Values shown are the means of two technical replicates. (c) ZmERF4 interacts and destabilizes ZmTPL1 in *Nicotiana benthamiana*. We performed co‐immunoprecipitation (Co‐IP) using anti‐myc antibody. Co‐IP shows interaction between ZmERF4 and ZmTPL1. Quantification of the ZmTPL1 protein signal in the input in presence of yellow fluorescent protein (YFP) or ZmERF4 was represented as FC ± SD. (d) Jsi1 interferes with the destabilization of ZmTPL1 mediated by ZmERF4 in *N. benthamiana*. Protein signal quantification of ZmTPL1, ZmERF4, and the different versions of Jsi1 in the input were normalized to the respective Rubisco signal (Ponceau staining). FC for each protein was expressed relative to the normalized protein value observed in ZmTPL1 co‐expressed with YFP (c) or Jsi1 and ZmERF4 (d). FC ± SD values represented in the bar graph are means of three biological replicates.

To test whether *U. maydis* is able to induce genes connected with the ERF branch in maize, we searched for genes orthologous to those induced by Jsi1 in *A. thaliana* and tested their expression by qRT‐PCR. We selected *ZmERF1*, *ZmERF1a*, *ZmERF2* and *ZmERF105* (which belong to the B3 group of the AP2/ERF family), *ZmERF12* (which belongs to the B1 group of the AP2/ERF family characterized as repressors of transcription) (Du *et al*., 2014), and *ZmACS6*, *ZmPR5* and *ZmOSM34*. Gene induction was evaluated during *U. maydis* infection at 4 and 6 dpi where Jsi1 expression is relatively high (Figs 4a, S1b). Most genes tested were found to be significantly induced at 4 and 6 dpi, with the exceptions of *ZmERF2* and *ZmOSM34* (which were not found to be induced) and *ZmERF1* (which was significantly induced only at 4 dpi) (Fig. 4a). These data indicate that *U. maydis* infection induces genes associated with the ERF branch in maize. To test whether Jsi1 is able to induce these genes in maize, we used detached maize leaves to express Jsi1 and Jsi1m under the 35S promoter via biolistic bombardment and evaluated gene expression 10 h after bombardment. *ZmERF1*, *ZmERF1a* and *ZmPR5* were induced by expression of Jsi1‐mCherry compared with Jsi1m‐mCherry in three replicates, whereas *ZmERF105* was induced in two replicates, and *ZmERF12* and *ZmACS6* were only induced in a single replicate (Fig. 4b). Our results demonstrate that Jsi1 induces genes connected with the ERF branch in maize, and the DLNxxP EAR motif, which is required for interaction with TPL/TPRs, plays an important role in this induction.

Regarding the GO category of SA‐response genes, we identified 39 upregulated genes, including *GRX480*, *ALD1* and *WRKY70*, which have been described as SA‐regulated genes (Li *et al*., 2004; Herrera‐Vásquez *et al*., 2014; Cecchini *et al*., 2015) (Fig. S6). In addition, we observed a cell‐death phenotype 3 d after expression of Jsi1 in *A. thaliana* plants (Fig. S5c). The cell‐death phenotype was also observed for prolonged expression of Jsi1 in *N. benthamiana* leaves 5 dpi (Fig. S5b). We further found that cell‐death induction correlates with the presence of the TPL/TPRs interaction EAR motifs, as shown in maize plants locally overexpressing Jsi1 after 10 dpi with the *Foxtail mosaic virus‐*based overexpression system and in *N. benthamiana* leaves (Fig. S5a,b) (Bouton *et al*., 2018). Activation of SA signaling could be due to recognition of the Jsi1–TPL interaction by the plant immune system, as previously reported (Gawehns, 2014), or the Jsi1–TPL interaction could interfere with the repressive activity of TPL‐interacting transcriptional regulators involved in suppressing the SA signaling pathway.

### Jsi1 promotes biotrophic susceptibility in *A. thaliana*

In order to assess whether the transcriptional changes observed in the JA/ET and SA signaling pathways correlated with changes in SA and JA hormone levels, we measured the levels of SA, JA and JA‐Ile, the bioactive form of JA, in *A. thaliana* shoots. The two *A. thaliana* XVE‐jsi1‐mCh lines expressing *jsi1* showed a significant increase in SA compared with the XVE‐mCh line, whereas we could not detect JA‐Ile or JA in either the XVE‐jsi1‐mCh lines or in the control (Fig. S3d). The lack of JA and JA‐Ile indicates activation of the ERF branch by Jsi1 is independent of the hormone itself. Activation of SA signaling should lead to repression of JA/ET signaling, as extensive crosstalk between these two signaling pathways has been reported (Caarls *et al*., 2015), and would increase resistance to biotrophic infection. To test how Jsi1 expression in *A. thaliana* impacts biotrophic susceptibility, we tested the XVE‐jsi1‐mCh and XVE‐mCh control lines after estradiol treatment for their susceptibility towards the hemibiotrophic pathogen *Pst* DC3000. We sprayed *jsi1*‐expressing and control lines with 150 nM estradiol to avoid the cell‐death phenotype associated with prolonged expression of *jsi1* during *Pst* DC3000 infection (Fig. S5c). *Jsi1*‐expressing lines were more susceptible to *Pseudomonas syringae* pv *tomato* (*Pst*) DC3000 infection than the control (Fig. 3c), indicating that activation of SA signaling in this context does not interfere with biotrophic susceptibility.

### Jsi1 may alter the repressing activity of ethylene response factors

The activation of JA/ET and SA signaling by Jsi1 could be a consequence of its interaction with TPL/TPR proteins, leading to interference with the repressive activity of endogenous DLNxxP‐containing transcriptional regulators. In *A. thaliana*, 67 transcriptional regulators were identified with a predicted DLNxxP motif from the B1 group of AP2/ERF and C2H2 families (Kagale *et al*., 2010). TFs that interact with TPL/TPRs are mainly negative regulators of transcription (Causier *et al*., 2012b). We therefore focused on genes that are upregulated in the Jsi1‐expressing lines that could be targets for DLNxxP‐containing TFs. Using previously available DAP‐seq data, we first determined genome‐wide putative direct target genes of six ERFs from the B1 subfamily, *ERF3*, *4*, *7*, *8*, *10* and *11*, and *ZAT10* from the *C2H2* family (O'Malley *et al*., 2016). Next, we compared the list of putative target genes for each TF with those genes upregulated in the *jsi1*‐expressing lines. Significant enrichment for each TF was evaluated by Fisher’s exact test (*P* < 0.05). Except for *ERF7* and *ZAT10*, transcriptional targets of the other ERFs were strongly enriched for genes de‐repressed by Jsi1. In total, 269 of the 915 genes upregulated by *jsi1* expression possess at least one transcription binding site (TBS) for an ERF (Fig. 3d; Table S4). GO‐term analysis of these upregulated genes showed 26 categories, 24 of which were previously identified in the GO‐term analysis of the Jsi1 RNA‐seq (Fig. S4b). In fact, from the 25 upregulated genes identified in the RNA‐seq that respond to ET, 72% possess TBSs for ERFs and only 33% of the 39 upregulated SA‐responsive genes possess TBSs for ERFs (Figs 3a, S6). This indicates that Jsi1 may regulate the expression of several ET‐responsive genes by altering the repressive activity of ERFs via interference with AtTPL/TPR proteins. Regarding the SA‐responsive genes, some of them might be regulated by ERFs. However, other unknown TFs whose interaction with TPL is altered by Jsi1 cannot be excluded.

To test whether Jsi1 can interfere with the interaction between ERFs and TPL/TPR proteins, we cloned *ZmERF4*, which was found to be induced in maize during *U. maydis* infection (Lanver *et al*., 2018) and possesses a DLNxxP and an LxLxL motif. We found that ZmERF4 interacted with ZmTPL1 both by Co‐IP in *N. benthamiana* and in a Y2H assay, where the second WD40 domain of ZmTPL1 was required for interaction (Figs 4c, S7a). Co‐expression of ZmERF4 with ZmTPL1 leads to destabilization of ZmTPL1, as protein amounts in the input were *c*. 80% lower than ZmTPL1 expressed with YFP (Fig. 4c). To test whether Jsi1_27641_ can interfere with ZmERF4‐mediated destabilization of ZmTPL1, we co‐expressed ZmTPL1 and ZmERF4 cloned in frame with Jsi1_27641_, Jsi1m_27641_, a version with a deletion at the N‐terminus including the EAR motif (Jsi1del_47641_), and an mCherry control. ZmERF4 was separated from the different Jsi1 versions by the porcine teschovirus‐1 2A co‐translational skipping motif to produce equimolar amounts of both proteins upon polycistronic expression (Kim *et al*., 2011). Jsi1_27641_ interferes with the destabilization of ZmTPL1 by ZmERF4, as ZmTPL1 protein levels in presence of Jsi1_27641_ are higher than ZmTPL1 protein levels in the presence of ZmERF4 with Jsi1m_27641_, Jsi1del_47641_, or the mCherry control (Fig. 4d). To test whether the interference of Jsi1 on ZmERF4 activity is due to a competition for its binding to ZmTPL1, we performed a Co‐IP experiment between ZmERF4 and ZmTPL1 in the presence of either Jsi1, Jsi1m_27641_ or Jsi1del_47641_. However, we did not find that Jsi1 competes with ZmERF4 for its binding to ZmTPL1, as the amount of ZmERF4 co‐precipitated with ZmTPL1 does not change when co‐expressed with the different versions of Jsi1 (Fig. S7b). These results suggest that Jsi1 might interfere with ZmERF4 activity via an unknown mechanism that involves TPL/TPR interaction and is associated with the upregulation of genes mainly connected with JA/ET signaling.

### Conserved EAR‐motif from different fungi effectors is responsible for interaction with corresponding TPL/TPR

**Fig. 5:**
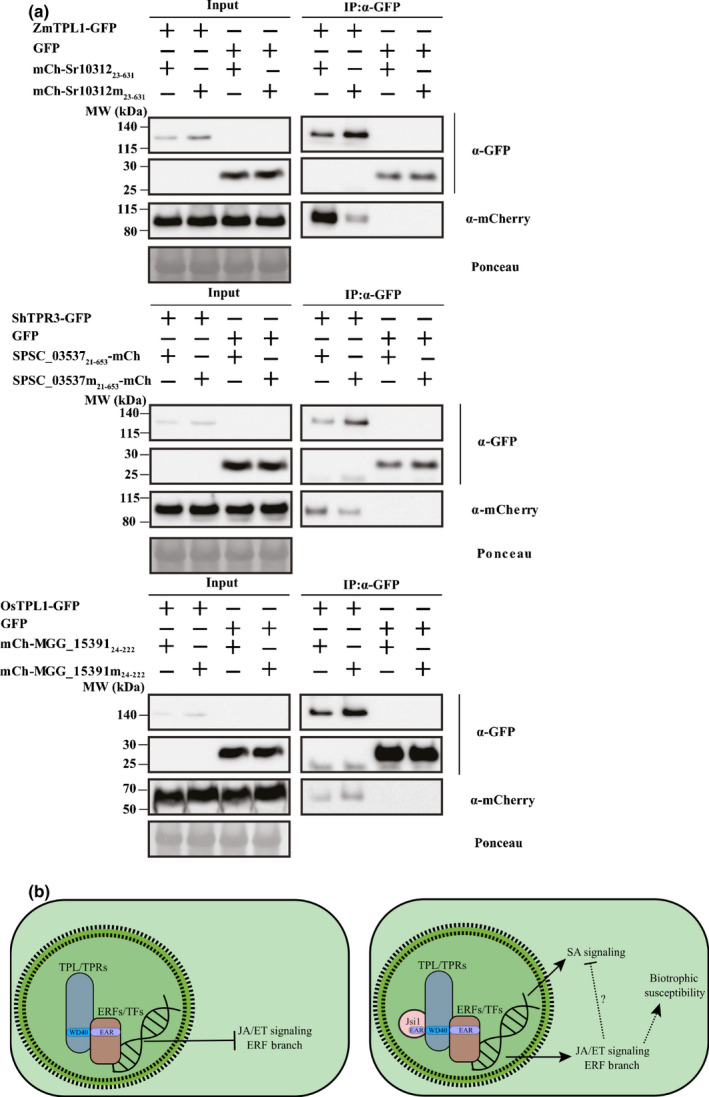
DLNxxP‐motif containing effectors of different fungal pathogens interacts with Topless (TPL). (a) We co‐infiltrated Sr10312, SPSC_03537, MGG_15391 and their versions mutated in the ethylene‐responsive element binding factor‐associated amphiphilic repression (EAR) motif (Sr10312m, SPSC_03537m and MGG_15391m) with their respective TPL proteins in *Nicotiana benthamiana* leaves. Co‐immunoprecipitated proteins were detected with anti‐GFP and anti‐mCherry antibodies. GFP, green fluorescent protein. (b) Jasmonate/Ethylene signaling inducer 1 (Jsi1) hijacks jasmonate/ethylene (JA/ET) signaling by interaction with TPL/Topless related (TPR) corepressors. Left panel: in the absence of the fungal Jsi1 effector, interaction between plant ethylene response factors (ERFs) and unknown transcription factors (TFs) possessing a DLNxxP with the second WD40 domain of TPL/TPR corepressor proteins may lead to repression of the ERF branch of the JA/ET signaling. Right panel: Jsi1 may interfere with the activity of ERFs and other unknown TFs, leading to activation of the ERF branch of the JA/ET signaling. Activation of salicylic acid (SA) signaling by Jsi1 could be due to activation of the plant immune system by recognition of the Jsi1‐TPL/TPRs interaction. On the other hand, SA signaling activation could also be due to Jsi1 interfering with the activity of ERFs. However, SA signaling cannot repress the ERF branch of the JA signaling as repressive ERF activity is blocked by the interaction between Jsi1 and TPL/TPRs. Therefore, a not fully activated SA defense pathway cannot lead to inactivation of the ERF branch, which may lead to biotrophic susceptibility *in planta*.

TPL/TPR proteins are highly conserved between different plant species (Causier *et al*., 2012a), so we asked whether DLNxxP‐motif‐containing effectors from various fungal pathogens with different hosts also use a similar strategy to manipulate TPL/TPR signaling. We performed a motif search analysis across published proteomes of plant pathogenic fungi to identify putative secreted effectors with a DLNxxP motif. We searched for effectors with a DLNxxP motif in the smut proteomes of *Ustilago hordei*, *Ustilago bromivora*, *S. scitamineum* and *S. reilianum* and identified additional effector candidates (Table 1). We performed the same search in plant pathogenic fungi with different lifestyles and from different fungal divisions. Based on sequence availability, we selected the obligate biotroph *Puccinia triticina* from the Basidiomycota division and from the Ascomycota division the biotrophic *Blumeria graminis*, the hemibiotrophic *F. oxysporum* and *M. oryzae*, and the necrotrophic pathogens *Botrytis cinerea*, *Sclerotinia sclerotiorum* and *Bipolaris maydis*. In all of the pathogens examined, with the exception of the necrotrophic pathogens, we found at least one predicted secreted protein possessing a DLNxxP motif after the secretion signal (Table 1), indicating that effectors possessing a DLNxxP motif are mainly found in pathogens with biotrophic and hemibiotrophic lifestyles. To test whether these effectors can interact with TPL, we selected *Sr10312* from *S. reilianum*, *SPSC_03537* from *S. scitamineum*, and *MGG_15391* from *M. oryzae*. As the *M. oryzae* effector belongs to a strain that infects rice (*Oryza sativa*) and *S. scitamineum* infects sugarcane (*Saccharum hybrid*), we cloned a rice TPL gene *OsTPL1* and a sugarcane TPL gene *Sh_TPR3* (Fig. S1c). We fused the effectors MGG_15391_24222_, Sr10312_23631_ and SPSC_03537_21653_ to mCherry and OsTPL1, ZmTPL1, and Sh_TPR3 to GFP. In addition, we mutated the DLNxxP motif of these effectors to AHNxxP (MGG_15391m_24222_, Sr10312m_23631_, and SPSC_03537m_21653_) to test the relevance of the EAR motif in the interaction with TPL. We performed Co‐IP assays by co‐expressing each effector and its mutated version with their respective host TPL protein in *N. benthamiana*. MGG_15391_24222_, Sr10312_23631_ and SPSC_03537_21653_ co‐immunoprecipitated with their respective TPL protein. Sr10312m_23631_ and SPSC_03537m_21653_ mutant versions did not pull down their respective TPL proteins as efficiently as their wild‐type versions, indicating that the EAR motif is responsible for their interaction with TPL (Fig. 5a). Thus, our data indicate that the DLNxxP motif from different fungal pathogens is responsible for interaction with the corresponding host TPL, suggesting that these pathogens have convergently evolved a strategy to manipulate the host‐signaling pathway by mimicking an endogenous host motif.

## Discussion

### Jsi1 interacts with the C‐terminus of TPL and hijacks jasmonate/ethylene signaling

Pathogens have developed diverse strategies to activate JA defense signaling, including producing bioactive forms or mimics of JA or effector proteins that activate JA signaling. Activation of JA signaling antagonizes SA signaling, promoting biotrophic susceptibility (Howe *et al*., 2018). In rice, the negative SA–JA signal crosstalk seems to be conserved (Yuan *et al*., 2007), but each hormone provides resistance to pathogens with different lifestyles (De Vleesschauwer *et al*., 2014). The SA–JA crosstalk also seems to be present in maize, but it has not been fully elucidated (Ziemann *et al*., 2018). Doehlemann *et al*. (2008) revealed that *U. maydis* infection of maize induces JA signaling and downregulates SA signaling. The induction of several members of the AP2/ERF family upon establishment of biotrophy by *U. maydis* suggests that induction of the ERF branch of the JA/ET defense signaling is beneficial for *U. maydis* infection. Nevertheless, its role in promoting biotrophic susceptibility in maize remains unknown. Here, we identified the *U. maydis* effector Jsi1 that activates JA/ET signaling. Jsi1 activates the ERF branch of the JA/ET defense signaling pathway by interacting with TPL/TPR proteins. In *A. thaliana*, Jsi1 induces the ERFs *ERF2*, *ERF5*, *ERF6* and *ERF107*, which are associated with resistance to necrotrophic pathogens and activation of the JA defense signaling pathway. Jsi1 also induces *PDF1.2*, further supporting the idea that Jsi1 activates the ERF branch of the JA signaling pathway. Finally, *A. thaliana* plants expressing Jsi1 are more susceptible to biotrophic infection, which also correlates with the activation of the ERF branch of the JA/ET signaling. In maize, overexpression of Jsi1 induces the expression of *ZmERF1*, *ZmERF1*, and *ZmPR5*, which were also induced during *U. maydis* infection. Taken together, these results demonstrate that Jsi1 contributes to the activation of the ERF‐branch of the JA/ET signaling pathway, which may promote fungal infection.

We have shown that Jsi1 interacts with TPL/TPRs via the second WD40 domain near their C‐terminus and that this interaction is dependent on the DLNxxP motif. This interaction induces the ERF branch of the JA/ET defense signaling pathway. ERF TFs with a DLNxxP motif were previously described to interact with AtTPL/TPRs (Causier *et al*., 2012b). For AtERF3 and AtERF4, the DLNxxP motif is essential for their repressive activity (Ohta *et al*., 2001). In addition, AtERF4 and AtERF9 can suppress the expression of *PDF1.2* and are negative regulators of resistance to necrotrophic pathogens, indicating that they act as negative regulators of the JA defense signaling (McGrath *et al*., 2005; Maruyama *et al*., 2013). The significant enrichment of TBSs of several ERFs with repressor activity in 269 out of the 915 genes upregulated upon Jsi1 induction and the ability of Jsi1 to interfere with the destabilization of ZmTPL1 by ZmERF4 show that ERFs with a DLNxxP motif are likely involved in repression of the ERF branch of the JA/ET signaling pathways.

Overexpression of ZmERF4 correlates with ZmTPL1 destabilization, suggesting that the repressive activity of ERFs is pernicious for the plant. Stabilization of ZmTPL1 in the presence of Jsi1 could indicate a protective effect, as we could not observe competition between Jsi1 and ZmERF4 for binding to ZmTPL1. It has been shown that TPL indirectly interacts with Histone Deacetylase 19 and both proteins are involved in transcriptional repression (Long *et al*., 2006). Therefore, Jsi1 may bind the C‐terminus of TPL and inhibit the interaction of other unknown repressor components required for ZmERF4’s repressive activity.

### Activation of salicylic acid signaling by Jsi1 expression does not lead to repression of jasmonate/ethylene signaling in *A. thaliana*

Jsi1 expression in *A. thaliana* leads to activation of the SA signaling pathway, as evidenced by the upregulation of several SA responsive genes and an increase in total SA levels. In addition, prolonged expression of Jsi1 leads to a cell‐death phenotype across the different plant species we tested, which may be connected to activation of SA signaling. Studying an effector function *in planta*, separated from the context of the rest of the effectome, may reveal complex responses derived from initial effector action and subsequent recognition responses by the plant immune system which would usually be counteracted by other effectors in the natural context (Thordal‐Christensen, 2020). It was reported that interaction between Six8, a *F. oxysporum* effector, and TPL leads to activation of SA defense signaling in *A. thaliana* (Gawehns, 2014). Therefore, the activity of Jsi1 on TPL may also trigger the plant immune system.

Another explanation is that the interaction between Jsi1 and TPL/TPR proteins could interfere with the repressive activity of TFs with a DLNxxP motif, leading to activation of the SA signaling pathway. SA‐signaling‐based inhibition of JA/ET signaling has been previously demonstrated (Caarls *et al*., 2015) and would lead to increased resistance towards biotrophic interactions. However, *A. thaliana* plants expressing Jsi1 are more susceptible to *Pst* DC3000 infection. Furthermore, *PDF1.2* and several ERFs related to activation of JA/ET signaling are upregulated by Jsi1, indicating that JA/ET signaling cannot be repressed by SA signaling. One explanation could be a potential role of EAR‐motif‐containing ERFs in mediating the SA repression of JA/ET signaling. It was previously reported that the promoter regions of genes induced by methyl jasmonate are enriched in a GCC‐box motif, the DNA binding motif of ERFs. In addition, it was shown that the GCC‐box is sufficient for transcriptional suppression by SA and that SA leads to degradation of ORA59, a positive regulator of the ERF branch (Van der Does *et al*., 2013). In summary, Jsi1 activates both JA/ET and SA‐responsive genes but SA antagonism on JA/ET signaling, which may be dependent on the ERFs–TPL/TPRs interaction cannot be exerted as a consequence of the interaction of Jsi1 with TPL/TPRs (Fig. 5b).

### Effectors of diverse biotrophic and hemibiotrophic fungi convergently evolved

Plant host proteins targeted by effectors are under selective pressure to evade manipulation by the pathogen. On the other hand, if central regulators like TPL/TPRs interact with many endogenous host proteins via a specific motif, like the DLNxxP motif, it becomes nearly impossible to mutate the binding sites without tremendous fitness costs to the plant. This is likely why effectors from diverse biotrophic and hemibiotrophic fungi, including *Sr10312* and *SPSC_03537* from *S. reilianum* and *S. scitamineum*, respectively, may have convergently evolved the DLNxxP motif to interfere with the transcriptional control of the co‐repressors from the Topless family.

## Author contributions

AD conceived the original research plan. AD and MD designed and coordinated the experimental work. DA, IF, JB, JM, K‐SC, KZ, MB, MD, LMSJ, IS, RB, SU and YP‐H contributed to the experimental work. AD, K‐SC and MD wrote the manuscript. MD and K‐SC contributed equally to this work.

## Supporting information


**Fig. S1** *jsi1* is part of effector cluster 2A and its deletion has no detectable contribution to the virulence of *U. maydis*.
**Fig. S2** Co‐IP assay showing that Jsi1 interacts with ZmTPL/TPRs in *Z. mays*.
**Fig. S3** Jsi1 induces the ERF‐branch in *A. thaliana*.
**Fig. S4** GO‐term analysis for biological process of genes differentially expressed in *A. thaliana* lines expressing Jsi1.
**Fig. S5** Prolonged expression of Jsi1 leads to a cell‐death phenotype in *Z. mays*, *N. benthamiana* and *A. thaliana*.
**Fig. S6** Heat map from RNA‐seq showing the GO category for SA responsive genes.
**Fig. S7** ZmERF4 binds to the C‐terminal of TPL but Jsi1 does not interfere with the ZmERF4 binding to ZmTPL1.
**Methods S1** Gene accession numbers, plasmid cloning, virulence assay in maize, phytohormone measurements, *Pseudomonas syringae* pv. *tomato* (*Pst*) DC3000 infection assay in *A. thaliana*, biolistic transformation of maize for virus‐mediated effector overexpression experiments.
**Table S1** Constructs used in this study.
**Table S2**. List of primers used for RT‐PCR.
**Table S3** Genes upregulated in *A* *.* *thaliana* XVE‐Jsi1 lines upon Jsi1 expression.
**Table S4** Gene upregulated in *A* *.* *thaliana* XVE‐Jsi1mCh lines upon Jsi1 expression nriched for ERFs transcription‐binding sites.
Please note: Wiley Blackwell are not responsible for the content or functionality of any Supporting Information supplied by the authors. Any queries (other than missing material) should be directed to the *New Phytologist* Central Office.
