# Supplementary material for: Ustilago maydis effector Jsi1 interacts with Topless corepressor, hijacking plant jasmonate/ethylene signaling
Source: New Phytol. 2021 Jan 3;229(6):3393–407. doi: 10.1111/nph.17116 (PMC8126959; doi:10.1111/nph.17116)
Supplement: Supplementary file 1 — Fig. S1 jsi1 is part of effector cluster 2A and its deletion has no detectable contribution to the virulence of U. maydis. Fig. S2 Co‐IP assay showing that Jsi1 interacts with ZmTPL/TPRs in Z. mays. Fig. S3 Jsi1 induces the ERF‐branch in A. thaliana. Fig. S4 GO‐term analysis for biological process of genes differentially expressed in A. thaliana lines expressing Jsi1. Fig. S5 Prolonged expression of Jsi1 leads to a cell‐death phenotype in Z. mays, N. benthamiana and A. thaliana. Fig. S6 Heat map from RNA‐seq showing the GO category for SA responsive genes. Fig. S7 ZmERF4 binds to the C‐terminal of TPL but Jsi1 does not interfere with the ZmERF4 binding to ZmTPL1. Methods S1 Gene accession numbers, plasmid cloning, virulence assay in maize, phytohormone measurements, Pseudomonas syringae pv. tomato (Pst) DC3000 infection assay in A. thaliana, biolistic transformation of maize for virus‐mediated effector overexpression experiments. Table S1 Constructs used in this study. Table S2. List of primers used for RT‐PCR. Table S3 Genes upregulated in A . thaliana XVE‐Jsi1 lines upon Jsi1 expression. Table S4 Gene upregulated in A . thaliana XVE‐Jsi1mCh lines upon Jsi1 expression nriched for ERFs transcription‐binding sites. Please note: Wiley Blackwell are not responsible for the content or functionality of any Supporting Information supplied by the authors. Any queries (other than missing material) should be directed to the New Phytologist Central Office. [file NPH-229-3393-s001.pdf]

New Phytologist **Supporting Information**

Article title: ***Ustilago maydis* effector Jsi1 interacts with Topless corepressor, hijacking plant JA/ET signaling**

Authors: **Martin Darino, Khong-Sam Chia, Joana Marques, David Aleksza, Luz Mayela Soto Jiménez, Indira Saado, Simon Uhse, Michael Borg, Ruben Betz, Janos Bindics, Krzysztof Zienkiewicz, Ivo Feussner, Yohann Petit-Houdenot and Armin Djamei**

Article acceptance date: 16 November 2020

The following Supporting Information is available for this article:

Methods S1 Gene accession numbers, plasmid cloning, virulence assay in maize, phytohormone measurements, *Pseudomonas syringae* pv. *tomato* (*Pst*) DC3000 infection assay in *A. thaliana*, biolistic transformation of maize for virus-mediated effector overexpression experiments

Fig. S1 *jsi1* is part of effector cluster 2A and its deletion has no detectable contribution to the virulence of *U. maydis*.

Fig. S2 Co-IP assay showing that Jsi1 interacts with ZmTPL/TPRs in *Z. mays*.

Fig. S3 Jsi1 induces the ERF branch in *A. thaliana.*

Fig. S4 GO-term analysis for biological process of genes differentially expressed in *A. thaliana* lines expressing Jsi1.

Fig. S5 Prolonged expression of Jsi1 leads to a cell-death phenotype in *Z. mays*, *N. benthamiana* and *A. thaliana*.

Fig. S6 Heat map from RNA-seq showing the GO category for SA responsive genes.

Fig. S7 ZmERF4 binds to the C-terminal of TPL but Jsi1 does not interfere with the ZmERF4 binding to ZmTPL1.

Table S1 Constructs used in this study.

Table S2. List of primers used for RT-PCR.Gene

Table S3 Genes upregulated in *A. thaliana* XVE-jsi1-mCh lines upon Jsi1 expression.

Table S4 Gene upregulated in *A. thaliana* XVE-jsi1-mCh lines upon Jsi1 expression enriched for ERFs transcription-binding sites.

Methods S1

Gene accession numbers

jsi1: UMAG_01236, *ZmTpl1*: GRMZM2G030422, *ZmTpl2*: GRMZM2G316967, *ZmTpl3*: GRMZM2G042992, *ZmERF1*: GRMZM2G381441, *ZmERF1a*: GRMZM2G123119, *ZmERF2*: GRMZM2G055180, *ZmERF4*: GRMZM2G310368, *ZmERF105*: GRMZM5G805505, *ZmERF12*: GRMZM2G002119, *ZmACS6*: GRMZM2G164405, *ZmPR5*: GRMZM2G402631, *ZmOSM34*: GRMZM2G136372, *OsTPL1*: Os03g0254700, *Sh_TPR3*: Sh_231H24

Plasmid cloning

Plasmid constructions were performed according to standard molecular cloning procedures (Ausubel et al., 1987; Sambrook & Russell, 2006) or using the GreenGate vector set and cloning conditions (Lampropoulos et al., 2013). All DNA manipulations were performed using the *Escherichia coli* MACH1 strain (Thermo Fisher Scientific, USA). Different modules were blunt-end ligated into the pJet vector (Thermo Fisher Scientific,Waltham, MA, USA) before further GreenGate cloning procedures. All modules used were either amplified by PCR or obtained from the published system. pUG plasmids for *U. maydis* transformation were adapting from p123 vector into GreenGate system compatible for ectopic insertion into *ip* locus (Aichinger *et al.,* 2003).

Virulence assay in maize

Three independent mutants in the *jsi1* locus (SG200Δ*jsi1*-1 to 3*)* were previously generated by Uhse *et al.,* (2018). To examine whether *jsi1* contributes to virulence, we assayed each of the mutant strain and SG200 strain on 7-day-old maize seedlings (100 plants per strain). We quantified symptom development 12 dpi according to Kämper *et al.,* (2006). Significant differences between *jsi1* mutant strains and SG200 were calculated using Fisher’s exact test. Multiple testing corrections were performed using the Benjamini-Hochberg algorithm. We repeated the experiment twice.

Phytohormone measurements

7-day-old *A. thaliana* plants were sprayed with a 5 µM β-estradiol solution and incubated for 6 hours. Phytohormone extraction was performed as described (Zienkiewicz *et al.,* 2020). Phytohormones were ionized in a negative mode and identified in a scheduled multiple reaction monitoring mode with an AB Sciex 4000 QTRAP® tandem mass spectrometer (AB Sciex, Framingham, MA, USA). We performed mass transitions as described (Iven *et al.*, 2012) with modifications specified in the table below. Five replicates per genotype were performed.

Mass transitions and corresponding conditions for determination of the phytohormones

**MRM Transitions**		**Analyte**	**DP [declustering potential]**	**EP**	**CE**
**Q1**	**Q3**			**[entrance potential]**	**[collision energy]**
137	93	SA	-25	-6	-20
141	97	D4-SA	-25	-6	-22
209	59	JA	-30	-4,5	-24
214	62	D5-JA	-35	-8,5	-24
322	130	JA-Ile/Leu	-45	-5	-28
325	133	D4-JA-Leu	-80	-4	-30

Pseudomonas syringae pv. *tomato* (*Pst*) DC3000 infection assay in *A. thaliana*

We performed *Pst* DC3000 infection assay as described (Liu *et al.*, 2015). Briefly, we sprayed 4-week-old *A. thaliana* plants with a solution of 150 nM β-estradiol, 12 hours before *Pst* DC3000 infiltration. We infiltrated leaves with a *Pst* DC3000 solution (OD_{600} = 0.0002). On the day of inoculation (0 dpi) and 2 dpi, leaf disks from six independent plants per genotype were collected. Bacterial proliferation was calculated as colony forming units (cfu) per cm^2 of leaf and visualized on a Log scale (cfu/cm^2). Statistical differences among the different genotypes were calculated using Tukey's HSD post-hoc test. The experiment was repeated three times.

Biolistic transformation of maize for virus-mediated effector overexpression experiments

Viral overexpression constructs were cloned via restriction enzyme XbaI and NotI which is based on the Foxtail mosaic virus (FoMV) described by Bouton et al., (2018). pFoMV-Jsi1$_{27-641}$, pFoMV-Jsi1m$_{27-641}$ and pFoMV-GFP were cloned by ligating insert fragment containing P19 silencing suppressor sequence followed by porcine teschovirus-1 2A (P2A) and CDS sequence (Jsi1$_{27-641}$, Jsi1m$_{27-641}$ or GFP) (Kim *et al.*, 2011). P2A sequence function as a unique ribosome skipping sequence and allow multiple proteins to express from the same RNA

transcript by putting it in between protein-coding sequences. 7-day-old maize seedlings were bombarded with the following viral constructs: pFoMV-Jsi1$_{27\text{-}641}$, pFoMV-Jsi1m$_{27\text{-}641}$ and pFoMV-GFP using 7 µg of each plasmid as described by Djamei et al., (2011). Seven days after bombardment, third leaves (non-bombarded leaves) were harvest and used for gene expression analysis. Gene expression was evaluated by qPCR following the same protocol described in the material and methods section. For trypan blue staining, we followed the protocol described by Fernández-Bautista et al., (2016) using 10 dpi maize infected leaves with the virus.

Fig. S1 *jsi1* is part of effector cluster 2A and its deletion has no detectable contribution to the virulence of *Ustilago maydis* (*U. maydis*). (**a**) Schematic representation of the *U. maydis* cluster 2a according to Kämper et al., (2006), *UMAG_01236* and *UMAG_01237* are in bold for being the only two genes of the cluster with a DLNxxP motif. (**b**) *UMAG_01236* is transcriptionally induced during infection. We extracted norm_count (normalized *U. maydis* reads counts) and FC (log2 (Fold change)) from Lanver *et al.,* (2018). (**c**) Phylogenetic tree of the Topless (TPL) protein orthologs. Scale bar indicates Jukes-Cantor distance. Bootstrap values are shown in branches. At, *Arabidopsis thaliana*; Zm, *Zea mays*; Os, *Oryza sativa*; Sh, *Saccharum hybrid*. (**d**) We used $Jsi1_{27\text{-}641}$ as prey and ZmTPL2, ZmTPL3, and N and C terminal regions from both ZmTPLs ($ZmTPL^{Nt}$ and $ZmTPL^{Ct}$) as bait in a Yeast two-Hybrid (Y2H) assay. The letters -L, -W and -H indicate medium lacking leucine, tryptophan and histidine, respectively. (**e**) Anti-TPL antibody was tested for specificity by using TPL protein from different plant species. We fused ZmTPL1, ZmTPL2 and ZmTPL3, OsTPL1 and ShTPR3 to GFP and independently infiltrated in *Nicotiana benthamiana* (*N. benthamiana*) leaves. We pulled-down the fused proteins from protein extracts using anti-GFP antibody. Western blot analysis with anti-GFP and TPL-specific antibody show that the TPL antibody can detect TPL proteins from different plants species. The red asterisk indicates full-length of the different TPL/TPRs fused to GFP and GFP protein. The black asterisk indicates the TPL/TPR proteins from *N. benthamiana* that were pulled-down by the TPL/TPRs fused to GFP. (**f**) Filamentation test on charcoal containing plates for all the strains that we used for virulence analysis and for confocal imaging. Filamentation on charcoal plates indicates that the tested strains are not affected in their ability to form hyphae, a morphological prerequisite for virulence. (**g**) Disease rating of Early Golden Bantam maize seedlings infected with *jsi1* mutants or progenitor strain SG200. We compared the virulence phenotype of three independent mutants with the virulence phenotype of the progenitor strain. Numbers above the columns indicate total number of plants infected in two independent experiments. Statistic differences between *jsi1* mutant strains and the SG200 progenitor were calculated using Fisher's exact test. Multiple testing corrections were performed using the Benjamini-Hochberg algorithm, (ns, not significant).

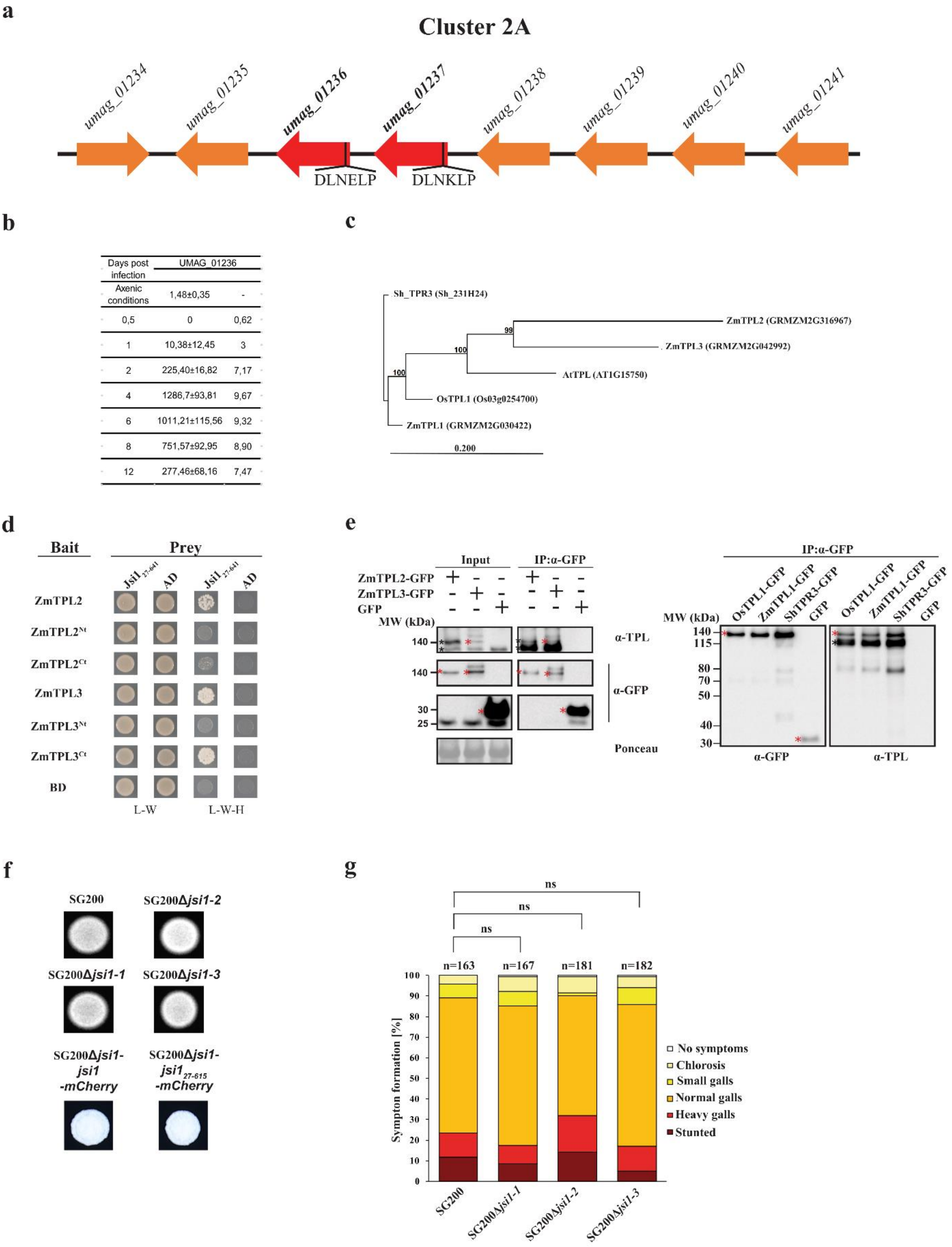

Days post infection	UMAG_01236	
Axenic conditions	1,48±0,35	-
0,5	0	0,62
1	10,38±12,45	3
2	225,40±16,82	7,17
4	1286,7±93,81	9,67
6	1011,21±115,56	9,32
8	751,57±92,95	8,90
12	277,46±68,16	7,47

Fig. S2 Co-immunoprecipitation **(**Co-IP) assay showing that Jsi1 interacts with ZmTPL/TPRs in *Zea mays*. We infected maize seedlings with each of the *Ustilago maydis* strains expressing Jsi1-3xHA, Jsi1m-3xHA and mCherry-3xHA and performed a Co-IP using anti-HA antibody. TPL-specific antibody shows that endogenous maize TPL/TPR proteins are co-purified with Jsi1-3xHA but not with Jsi1m-3xHA nor mCherry-3xHA. Red asterisks indicate the full-length proteins of Jsi1-3xHA, Jsi1m-3xHA and mCherry-3HA. Ponceau staining was used as loading control. Uncropped blots from Figure1**c** **(a)** and an independent second replicate of the experiment (**b**). mCherry protein was developed after 15 or 30 min of exposure while Jsi1 and Jsi1m were developed after 3 to 4 hours of exposure.

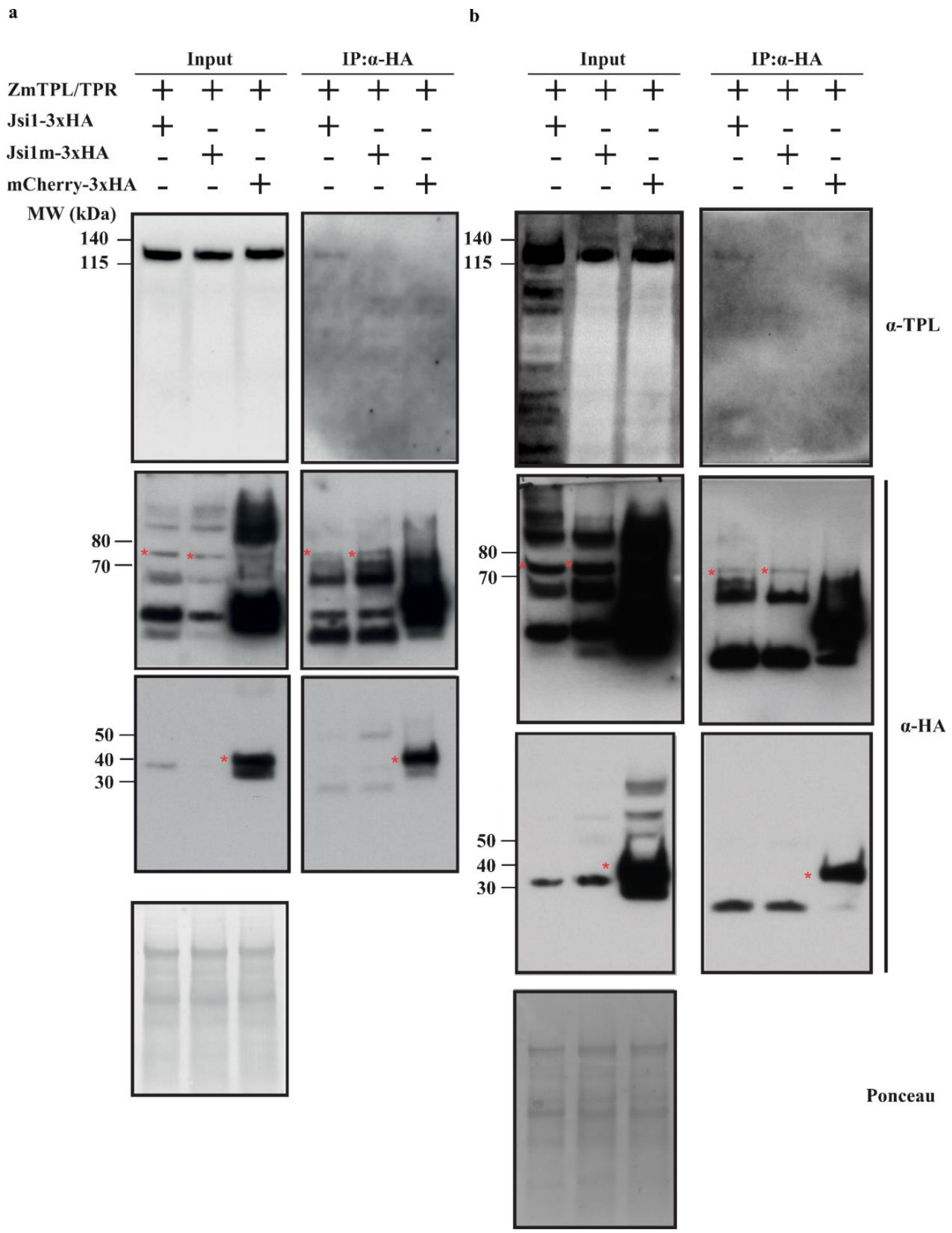

Fig. S3 Jsi1 induces the ethylene response factor (ERF) branch in *Arabidopsis thaliana* (*A. thaliana*). (**a**) We used $Jsi1_{27\text{-}641}$ as prey and AtTPL, AtTPR1, AtTPR2 and AtTPR4 as bait to test if Jsi1 can interact with the AtTPL/AtTPR proteins in Y2H assay. The letters -L, -W and -H indicate medium lacking leucine, tryptophan and histidine, respectively. (**b**) Estradiol inducible XVE-jsi1-mCh 1 and 2, and control XVE-mCh Arabidopsis-lines. We treated *A. thaliana* seedlings with 5µM of β-estradiol for 6 hours. We determined the expression levels of the proteins, Jsi1-mCherry or mCherry alone, using anti-mCherry antibody. Anti-Histone 3 antibody (Abcam) was used as loading control. Asterisk indicates full-length protein. (**c**) Principal Coordinates Analysis (PCA) demonstrates that RNA-seq libraries from induced XVE-jsi1-mCh lines grouped together while libraries from XVE-mCh control line either treated with or without β-estradiol cluster together separately from the Jsi1 lines. (**d**) Total Salicylic acid content in shoots of fresh *A. thaliana* seedlings. We sprayed XVE-jsi1-mCh 1 and 2 and XVE-mCherry lines with 5µM of β-estradiol to induce jsi1-mCh and mCherry expression, respectively. Each bar represents mean±SD of five replicates. Significant differences between *A. thaliana* XVE-jsi1-mCh and XVE-mCherry lines were calculated using Kruskal Wallis test followed by Dunn multiple comparison test (*P* value < 0.05). Different letters above the bars indicate statistically significant differences among the lines. (**e**) qPCR validation of genes from the RNA-seq. Gene induction was represented for each Jsi1 line. Fold Change ± Standard Deviation (FC±SD) is relative to the expression in the control mCherry line and normalized to the actin RNA expression values for three independent replicates. We evaluated statistically significant differences between gene expression in each Jsi1 lines related to the control mCherry line using ANOVA followed by Dunnett's multiple compared test (****$P < 0.0001$, ***$P < 0.001$, **$P < 0.01$, *$P < 0.05$, ns, not significant).

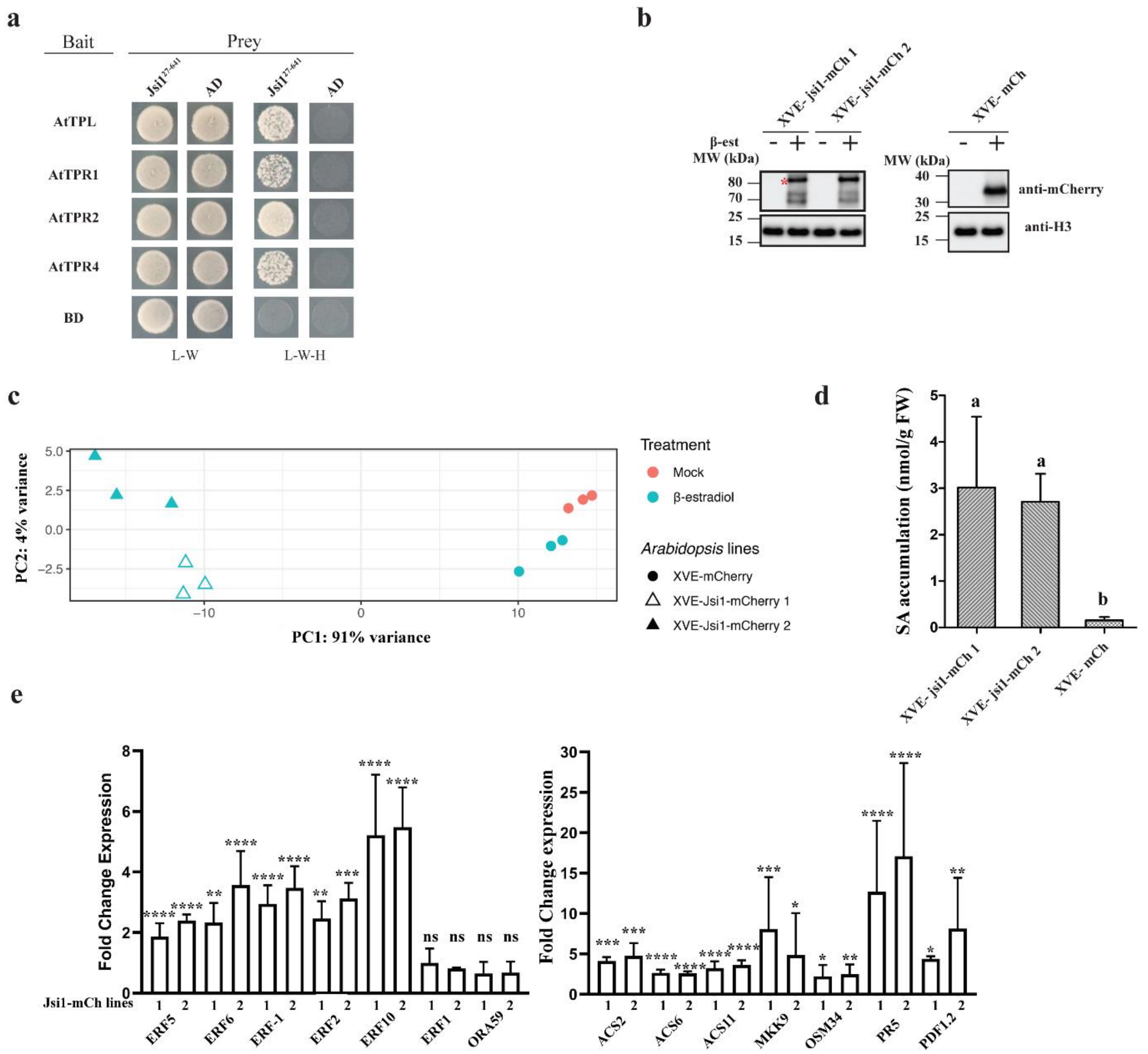

a
Bait
Prey
AtTPL
AtTPR1
AtTPR2
AtTPR4
BD
L-W
L-W-H
b
XVE- jsi1-mCh 1
XVE- jsi1-mCh 2
XVE- mCh
β-est
MW (kDa)
anti-mCherry
anti-H3
c
PC1: 91% variance
PC2: 4% variance
Treatment
Mock
β-estradiol
Arabidopsis lines
XVE-mCherry
XVE-Jsi1-mCherry 1
XVE-Jsi1-mCherry 2
d
SA accumulation (nmol/g FW)
e
Fold Change Expression
Jsi1-mCh lines
ERF5
ERF6
ERF-1
ERF2
ERF10
ERF1
ORA59
Fold Change expression
ACS2
ACS6
ACS11
MKK9
OSM34
PR5
PDF1.2

Fig. S4 GO-term analysis for biological process of genes differentially expressed in *Arabidopsis thaliana* lines expressing Jsi1. (**a**) Go-term analysis for the 1,090 differentially expressed genes in lines expressing Jsi1. (**b**) GO analysis for the 269 genes found to be enriched in transcription factor binding sites for Ethylene response factors (ERFs) protein. Percentage of genes were calculated for each category related to the total number of genes. All GO categories showed P value <0.05. GO categories with more than 20 genes were represented in the graph.

a

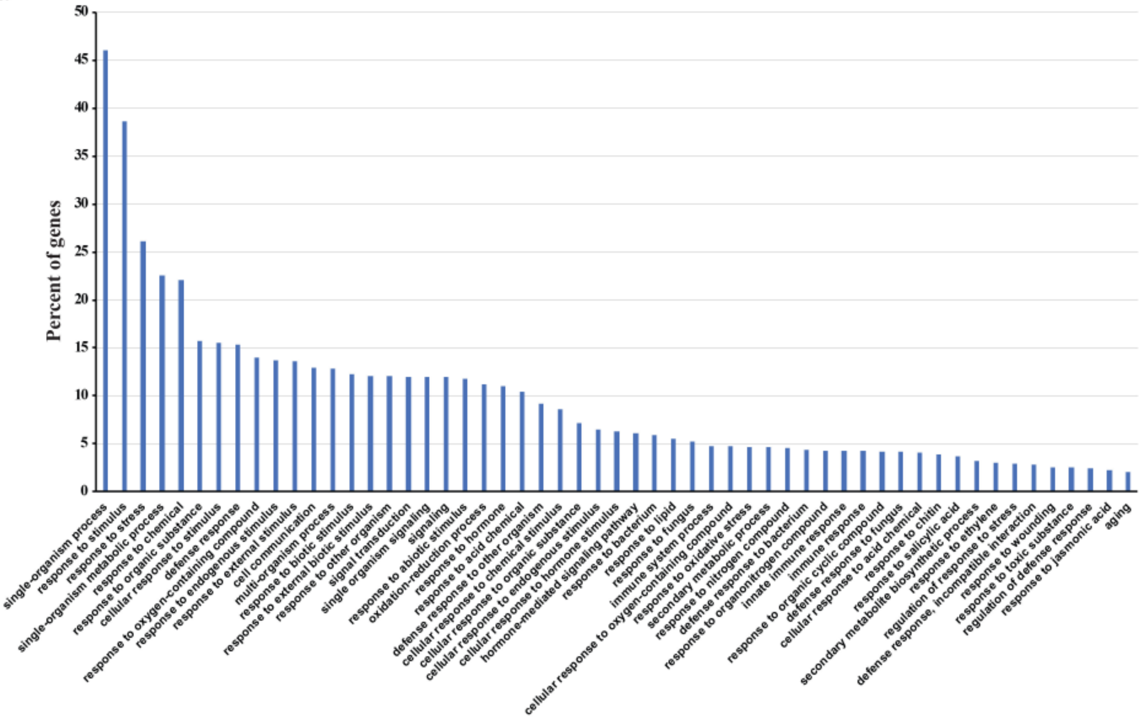

b

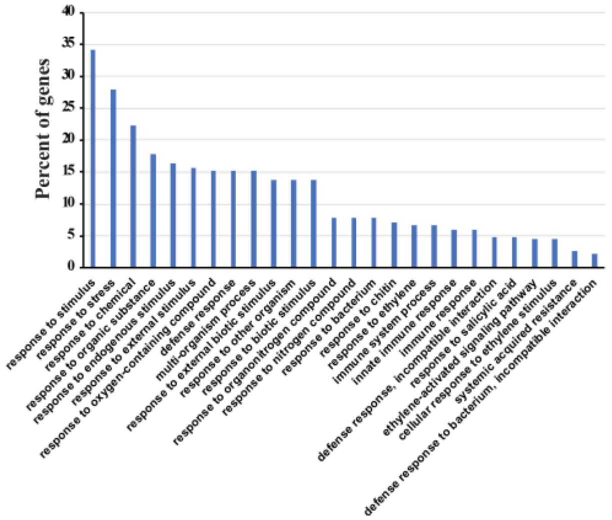

Fig. S5 Prolonged expression of Jsi1 leads to a cell-death phenotype in *Zea mays*, *Nicotiana benthamiana* (*N. benthamiana*) and *Arabidopsis thaliana* (*A. thaliana*). (**a**) **Left panel**, Trypan blue assay on detached maize leaves expressing pFoMV-GFP, pFoMV-Jsi1 and pFoMV-Jsi1m at 10 days post-infection (dpi) (n=2). Yellow arrow indicates cell-death in the leaf expressing Jsi1. Bars 1 cm. **Right panel**, *P19* genes expression levels in maize leaves infected with virus carrying pFoMV-GFP, pFoMV-Jsi1 and pFoMV-Jsi1m after 7 dpi. Fold Change ± Standard Deviation (FC±SD) is relative to the expression in maize plants without virus infection and normalized to the cyclin-dependent kinase (CDK) RNA expression values. Values shown are the means of two replicates. Statistically significant differences between genes expressed in maize plants infected with pFoMV-GFP and pFoMV-Jsi1 or pFoMW-Jsi1m were calculated using Mann-witness test (*****, *P* value <0.05). (**b**) **Left panel**, Cell-death phenotype by Jsi1 in *N. benthamiana* leaves at 5 dpi. Bars 1.5 cm. **Right panel**, Co-IP showing association only between Jsi1 and ZmTPL1 while Jsi1m weakly interacts with ZmTPL1 upon co-expression in *N. benthamiana*. Co-immunoprecipitated proteins were detected with anti-myc and anti-mCherry antibodies. (**c**) We sprayed 4-weeks old *A. thaliana* plants with 150nM and 5µM of β-estradiol. We took pictures at 1-, 2- and 3- days post β-estradiol treatment (dpt). White arrows indicate visible cell-death in *A. thaliana* lines expressing Jsi1. Bars 2 cm.

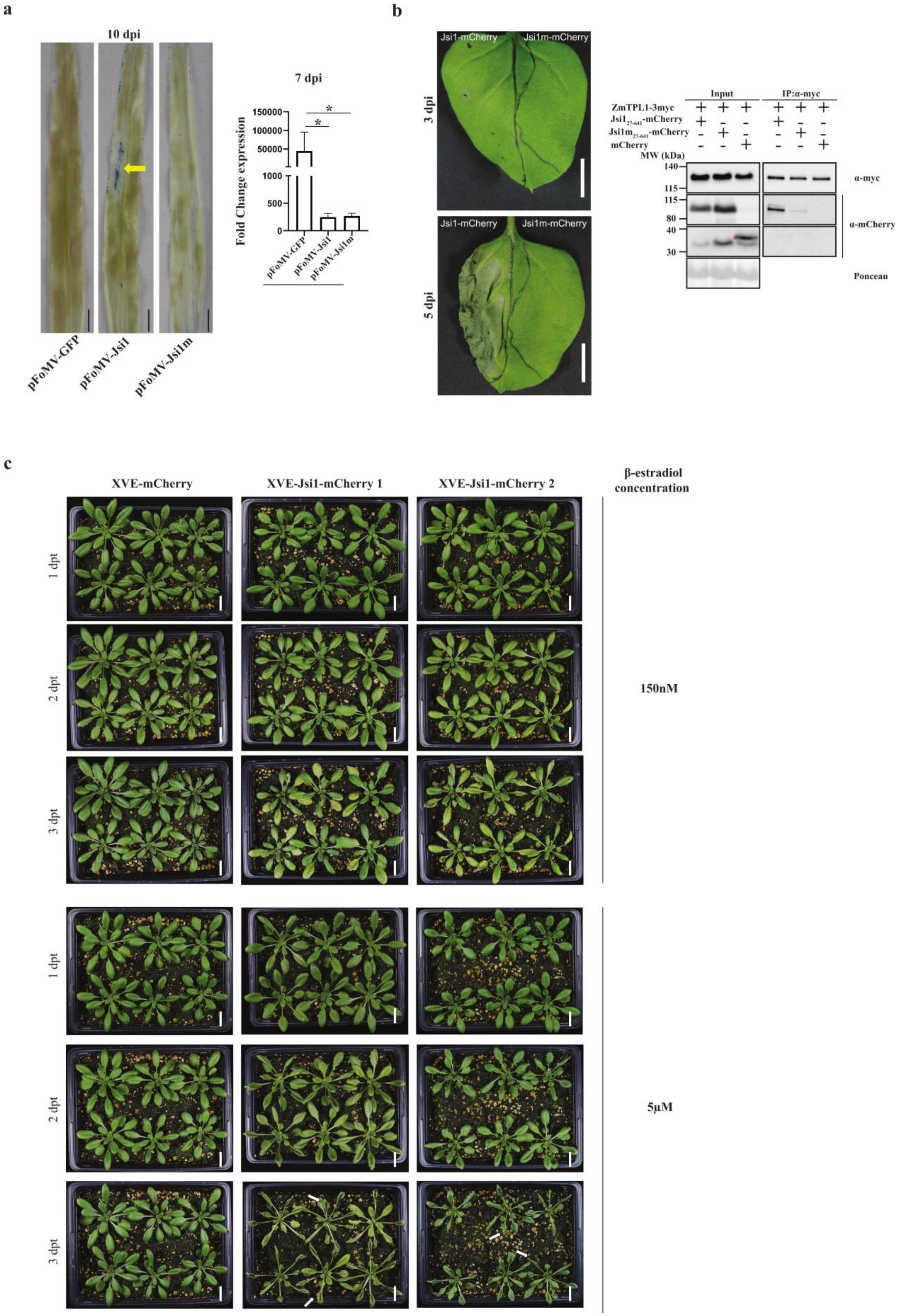
a
10 dpi
pFoMV-GFP
pFoMV-Jsi1
pFoMV-Jsi1m
7 dpi
Fold Change expression
150000
100000
50000
1000
500
0
*
*
pFoMV-GFP
pFoMV-Jsi1
pFoMV-Jsi1m
b
Jsi1-mCherry
Jsi1m-mCherry
3 dpi
5 dpi
Input
IP:α-myc
ZmTPL1-3myc
Jsi1$_{27-641}$-mCherry
Jsi1m$_{27-641}$-mCherry
mCherry
MW (kDa)
140
115
115
80
40
30
α-myc
α-mCherry
Ponceau
c
XVE-mCherry
XVE-Jsi1-mCherry 1
XVE-Jsi1-mCherry 2
β-estradiol concentration
1 dpt
2 dpt
3 dpt
150nM
1 dpt
2 dpt
3 dpt
5μM

Fig. S6 Heat map from RNA-seq showing the GO category for SA responsive genes. [a]Genes enriched in transcription binding sites (TBS) from ERF TFs with repression activity. Numbers under lines represent independent biological replicates for each line and treatment with or without ß-estradiol.

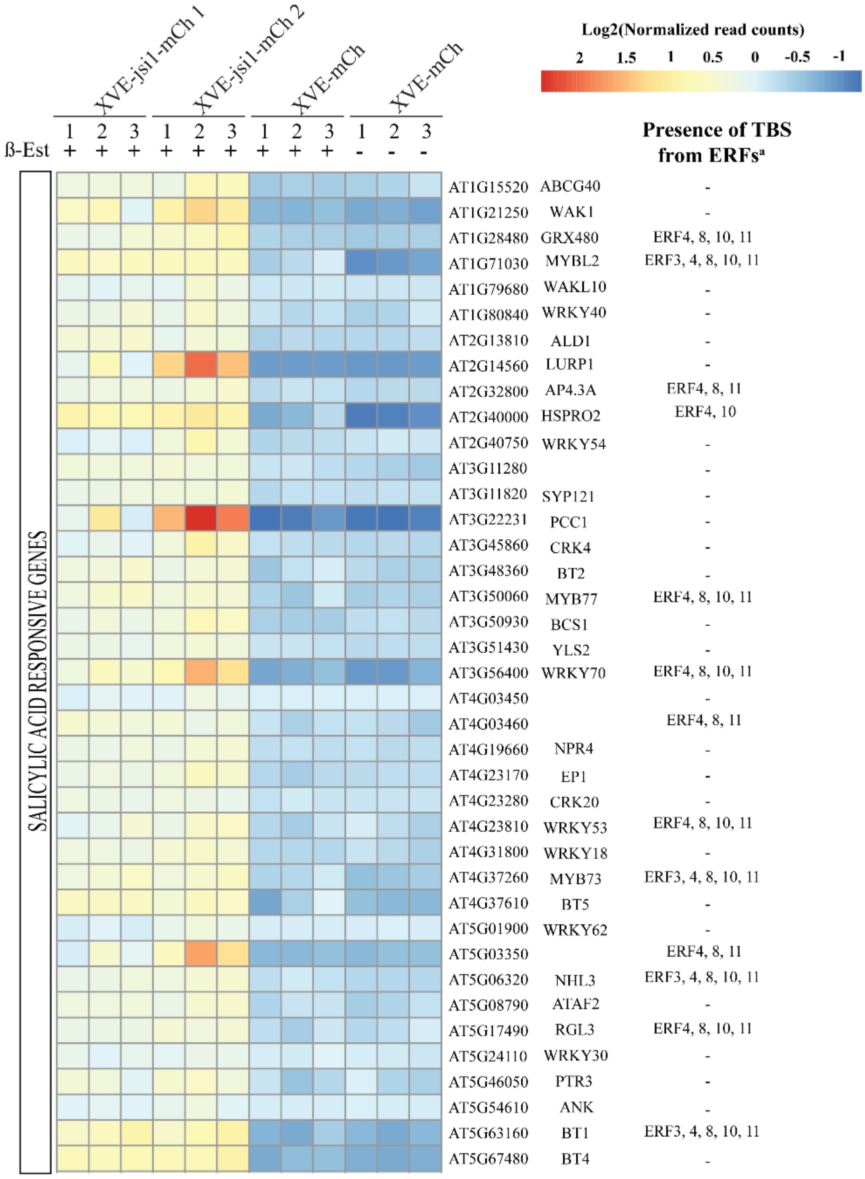

Fig. S7 ZmERF4 binds to the C-terminus of TPL but Jsi1 does not interfere with the ZmERF4 binding to ZmTPL1. (**a**) We tested ZmERF4 for interaction with full-length ZmTPL1, N and C-terminal parts of ZmTPL1 in Yeast two-Hybrid (Y2H) assay. The letters -L, -W and -H indicate medium lacking leucine, tryptophan and histidine, respectively. (**b**) Co-immunoprecipitation (Co-IP) showing that Jsi1 does not interfere with the ZmERF4 binding to ZmTPL1. Co-IP was performed using anti-myc antibody. Quantification of pulled-down proteins signal of ZmERF4 and the different Jsi1 versions were normalized to their respective pulled-down ZmTPL1 protein signal. Then, Fold Change (FC) for each protein was expressed relative to the value observed in ZmTPL1 co-expressed with Jsi1 and ZmERF4. FC±SD is a mean of three biological replicates, error bar represents standard deviation

a

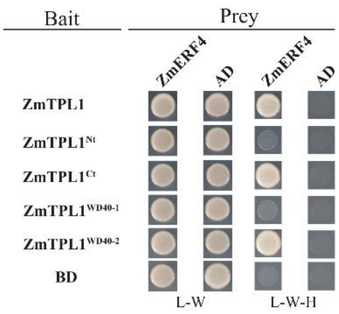

b

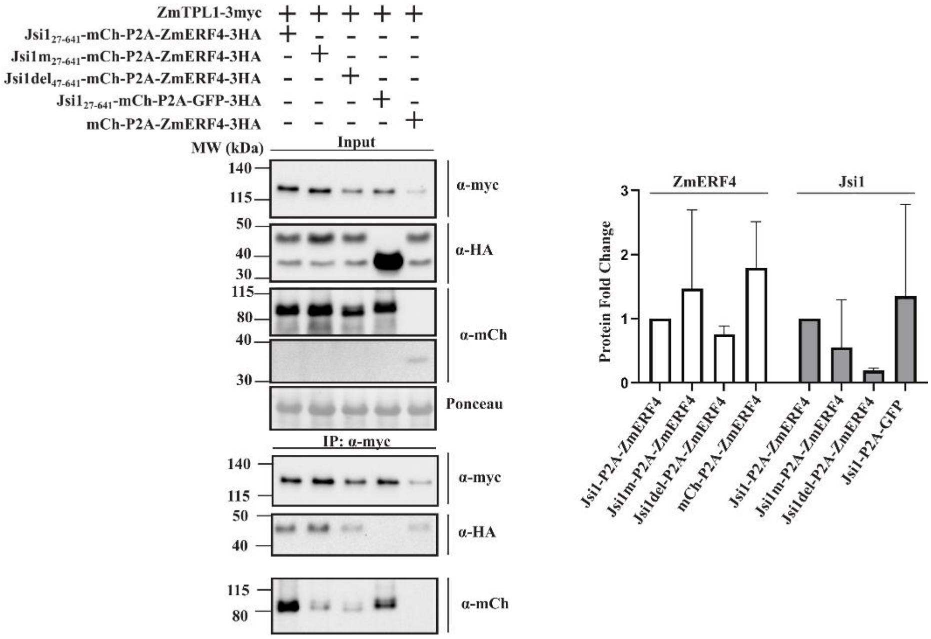

Table S1 Constructs used in this study

Construct	Resistance	Description
pGG187[1]-pADH1-GAL4BD-Myc-ZmTPL1-adh1T	Tryptophan, Spectinomycin	Bait constructs (GAL4BD) used for Y2H assay. Yeast strain AH109(MATa)
pGG187-pADH1-GAL4BD-Myc-ZmTpl1$^{Nt}_{1\text{-}218}$-adh1T	Tryptophan, Spectinomycin	
pGG187-pADH1-GAL4BD-Myc-ZmTpl1$^{Ct}_{215\text{-}1128}$-adh1T	Tryptophan, Spectinomycin	
pGG187-pADH1-GAL4BD-Myc-ZmTpl1$^{WD40\text{-}1}_{215\text{-}721}$-adh1T	Tryptophan, Spectinomycin	
pGG187-pADH1-GAL4BD-Myc-ZmTpl1$^{WD40\text{-}2}_{722\text{-}1128}$-adh1T	Tryptophan, Spectinomycin	
pGG187-pADH1-GAL4BD-Myc-eYFP-adh1T	Tryptophan, Spectinomycin	
pGG187-pADH1-GAL4BD-Myc-ZmTPL2-adh1T	Tryptophan, Spectinomycin	
pGG187-pADH1-GAL4BD-Myc-ZmTpl2$^{Nt}_{1\text{-}217}$-adh1T	Tryptophan, Spectinomycin	
pGG187-pADH1-GAL4BD-Myc-ZmTpl2$^{Ct}_{213\text{-}1082}$-adh1T	Tryptophan, Spectinomycin	
pGG187-pADH1-GAL4BD-Myc-ZmTPL3-adh1T	Tryptophan, Spectinomycin	
pGG187-pADH1-GAL4BD-Myc-ZmTpl3$^{Nt}_{1\text{-}219}$-adh1T	Tryptophan, Spectinomycin	
pGG187-pADH1-GAL4BD-Myc-ZmTpl3$^{Ct}_{216\text{-}1141}$-adh1T	Tryptophan, Spectinomycin	
pGG187-pADH1-GAL4BD-Myc-AtTPL-adh1T	Tryptophan, Spectinomycin	
pGG187-pADH1-GAL4BD-Myc-AtTPR1-adh1T	Tryptophan, Spectinomycin	
pGG187-pADH1-GAL4BD-Myc-AtTPR2-adh1T	Tryptophan, Spectinomycin	
pGG187-pADH1-GAL4BD-Myc-AtTPR4-adh1T	Tryptophan, Spectinomycin	
pGG446[1]-pADH1-Jsi1$_{27\text{-}641}$-GAL4AD-HA-adh1T	Leucine, Spectinomycin	Prey constructs (GAL4AD) used for Y2H assay. Yeast strain Y187 (MATα).
pGG446-pADH1-Jsi1m$_{27\text{-}641}$-GAL4AD-HA-adh1T	Leucine, Spectinomycin	
pGG446-pADH1-GAL4AD-HA-eYFP-adh1T	Leucine, Spectinomycin	
pGADT7-pADH1-GAL4AD-HA-ZmERF4-adh1T	Leucine, Ampicillin	

Continuation of **Table S1**

Construct	Resistance	Description
pGGZ001-35S-Ω-ZmTPL1-3xMyc-Ubq10T-BastaR	Basta, Spectinomycin	
pGGZ001-35s-Ω-ZmTPL1-eGFP-Ubq10-BastaR	Basta, Spectinomycin	
pGGZ001-35s-Ω-ZmTPL2-eGFP-Ubq10-BastaR	Basta, Spectinomycin	
pGGZ001-35s-Ω-ZmTPL3-eGFP-Ubq10-BastaR	Basta, Spectinomycin	
pGGZ001-35S-Ω-Jsi1$_{27\text{-}641}$-mcherry-Ubq10T-BastaR	Basta, Spectinomycin	
pGGZ001-35S-Ω-Jsi1m$_{27\text{-}641}$-mcherry-Ubq10T-BastaR	Basta, Spectinomycin	
pGGZ001-35S-Ω-mcherry-3xHA-Ubq10T-HygR	Hygromycin, Spectinomycin	
pGGZ001-35S-Ω-ZmERF4-3xHA-Ubq10T-BastaR	Basta, Spectinomycin	
pGGZ001-35S-Ω-YFP-3xHA-Ubq10T-HygR	Hygromycin, Spectinomycin	
pGGZ001-35S-Ω-Jsi1$_{27\text{-}641}$-mcherry-P2A-ZmERF4-3xHA-Ubq10T-BastaR	Basta, Spectinomycin	
pGGZ001-35S-Ω-Jsi1m$_{27\text{-}641}$-mcherry-P2A-ZmERF4-3xHA-Ubq10T-BastaR	Basta, Spectinomycin	Constructs used for Co-IP experiments performed in *N. benthamiana*. Agrobacterium strain GV3101 pMP90 pSoup
pGGZ001-35S-Ω-Jsi1del$_{47\text{-}641}$-mcherry-P2A-ZmERF4-3xHA-Ubq10T-BastaR	Basta, Spectinomycin	
pGGZ001-35S-Ω-Jsi1$_{27\text{-}641}$-mcherry-P2A-GFP-3xHA-Ubq10T-BastaR	Basta, Spectinomycin	
pGGZ001-35S-Ω-mcherry-P2A-ZmERF4-3xHA-Ubq10T-BastaR	Basta, Spectinomycin	
pGGZ001-35S-Ω-OsTPL1-GFP-Ubq10T-BastaR	Basta, Spectinomycin	
pGGZ001-35S-GFP-Ubq10T-BastaR	Basta, Spectinomycin	
pGGZ001-35S-Ω-myc-mCherry-MGG_15391$_{24\text{-}222}$-Ubq10T-BastaR	Basta, Spectinomycin	
pGGZ001-35S-Ω-myc-mCherry-MGG_15391m$_{24\text{-}222}$-Ubq10T-BastaR	Basta, Spectinomycin	
pGGZ001-35S-Ω-mCherry-Sr10312$_{23\text{-}631}$-Ubq10T-BastaR	Basta, Spectinomycin	
pGGZ001-35S-Ω-mCherry-Sr10312m$_{23\text{-}631}$-Ubq10T-BastaR	Basta, Spectinomycin	
pGGZ001-35S-Ω-SPSC_03537$_{21\text{-}653}$-mCherry-Ubq10T-BastaR	Basta, Spectinomycin	
pGGZ001-35S-Ω-SPSC_03537m$_{21\text{-}653}$-mCherry-Ubq10T-BastaR	Basta, Spectinomycin	
pGGZ001-35S-Ω-ShTPR3-GFP-Ubq10T-BastaR	Basta, Spectinomycin	

Continuation of **Table S1**

Construct	Resistance	Description
pUG[2]-P$_{cmu1}$-Jsi1-3xHA-nosT	Carboxin, Spectinomycin	Constructs used for Co-IP experiment in maize. SG200 strain
pUG-P$_{cmu1}$-Jsi1m-3xHA-nosT	Carboxin, Spectinomycin	
pUG-P$_{cmu1}$-SP$_{cmu1}$-mCherry-3xHA-nosT	Carboxin, Spectinomycin	
pGGZ001-35S-Ω-GFP-nls-Ubq10T-HygR	Hygromycin, Spectinomycin	Constructs used for Biolistic transformation of maize
pGGZ001-35S-Ω-ZmTPL1-GFP-Ubq10T-BastaR	Basta, Spectinomycin	
pUG-P$_{otef}$-Jsi1-3xHA-tNOS	Carboxin, Spectinomycin	Construct used for secretion experiment in axenic culture. AB33 strain
pUG-P$_{cmu1}$-Jsi1-mCherry-HA-tNOS	Carboxin, Spectinomycin	Constructs used for secretion experiment *in planta*. SG200Δ*jsi1* strain
pUG-P$_{cmu1}$-Jsi1$_{27\text{-}641}$-mCherry-HA-tNOS	Carboxin, Spectinomycin	
pGGZ001-35s:XVE-Jsi1$_{27\text{-}641}$-mCherry-Myc-Ubq10-BastaR	Basta, Spectinomycin	Constructs used for *A.thaliana* transformation
pGGZ001-35s:XVE-mCherry-Myc-Ubq10-BastaR	Basta, Spectinomycin	
pFoMV-P19-P2A- Jsi1$_{27\text{-}641}$-myc	Kanamycin	Constructs used for viral infection in maize
pFoMV-P19-P2A- Jsi1m$_{27\text{-}641}$-myc	Kanamycin	
pFoMV-P19-P2A- GFP-myc	Kanamycin	

[1] pGG187 and pGG446 are plasmids designed for Golden gate cloning using the pGBKT7 and pGADT7 (Clontech®) as backbones respectively.

[2] pUG is a vector designed from the p123 vector for Golden gate cloning (Lampropoulos *et al.*, 2013).

Table S2 List of primers used for RT-PCR

Primer	Gene ID	5'-3' sequence
ERF-1_F	AT4G17500	TTGCGGCGGAGATTAGAGAC
ERF-1_R		ATTCAACAAAGCGCGGGAAC
ERF1_F[2]	AT3G23240	TCAGAAGACCCCAAAAGCTCCTCA
ERF1_R[2]		TTGATCACCGCTCCGTGAAGTTAG
ERF2_F	AT5G47220	TTCACTGCCGTCTCCGTAAC
ERF2_R		GCGGTTAAAGTCGAGCCAAC
ERF5_F	AT5G47230	GGTGGAGAGACGTTTCCGTT
ERF5_R		ACCAAACGGTGGATGAGGAG
ERF6_F	AT4G17490	TTGTACAGGCCACGACCATC
ERF6_R		GGCGATTCTGAATTTCCCGC
ERF10_F	AT1G03800	CCCCCGGAGATAACATGTCG
ERF10_R		CAACCTTTCCGAGACGGTGA
ORA59_F	AT1G06160	CTCTGCTTCTACAATTTTTATG
ORA59_R		CTACACATCTATACATGTTTCC
ACS2_F[5]	AT1G01480	CATGTTCTGCCTTGCGGATC
ACS2_R[5]		ACCTGTCCGCCACCTCAAGT
ACS6_F	AT4G11280	ACGAGACGGTTGCTTTCTGT
ACS6_R		AGCGGGTTTGAAGGATTGGT
ACS11_F	AT4G08040	TTACCATGGCTTGCCAGCTT
ACS11_R		TCGTTAGCCGAGGTTGATCC
MKK9_F	AT1G73500	CGCCGTACAGATTCACCGTA
MKK9_R		TTTAGCGAATCCGGCGAGTT
OSM34_F	AT4G11650	CACCAAACACGTTGGCTGAG
OSM34_R		TGTCCGTTTATGTCTGCGGT
PR5_F	AT1G75040	CATCACCCACAGCACAGAGA
PR5_R		ACGGTGGTAGGGCAATTGTT
PDF1.2_F[1]	AT5G44420	TTGCTGCTTTCGACGCA
PDF1.2_R[1]		TGTCCCACTTGGCTTCTCG
actin2_F[3]	AT3G18780	TCGGTGGTTCCATTCTTGCT
actin2_R[3]		GCTTTTTAAGCCTTTGATCTTGAGAG

Continuation of **Table S2**

Primer	Gene ID	5'-3' sequence
ZmERF1_F		CAGAGGAGCAGACCATGTCC
ZmERF1_R	GRMZM2G381441	GTTCTTGACTTGTGCGTGCC
ZmERF1a_F		TAACCCATAGCGAATGGCGG
ZmERF1a_R	GRMZM2G123119	CTGAACTCGATCCCCAACCC
ZmERF2_F		GCCGTTCAACTCGATCACTTCTTTC
ZmERF2_R	GRMZM2G055180	GGAACGACCGAGAGGGAGAG
ZmERF105_F		TGGTCCCGATGGTGAACAAA
ZmERF105_R	GRMZM5G805505	GTCTCCCAGTACTGCTCCGA
ZmERF12_F		CTACTCCGTGGCATTCCGTC
ZmERF12_R	GRMZM2G002119	AATCCGATCGAGCATCAGCA
ZmACS6_F		GCCTGAACGCGTGCTAATTT
ZmACS6_R	GRMZM2G164405	CAGGGACAGTTGGTTCTCGG
ZmPR5_F		GCCGGAATAGGCTCTGCATT
ZmPR5_R	GRMZM2G402631	ACGAAGCACGCACACAAATC
ZmOSM34_F		TGATCGACGGCTACAACCTG
ZmOSM34_R	GRMZM2G136372	TTCGCAAACAAAGAAGTTGGCT
P19_F		TCCTTGGATCTTGGACGGGA
P19_R		GGGCAAGCTGTAGCAGTTCT
ZmCDK_F[4]		CCGTCATCGCCTCACGAAGAG
ZmCDK_R[4]	GRMZM2G149286	AGAGCCTGCCTTACGGAATTGG

[1, 2, 3, 4 and 5] primers pairs from Brown *et al*., (2003), McGrath *et al*., (2005), Zhang *et al*., (2011), Lin *et al*., (2014) and Schellingen *et al*., (2014), respectively.

Table S3 Genes upregulated in *A. thaliana* XVE-Jsi1 lines upon Jsi1 expression.

		Fold change			*P* value		
		XVE:	McGrath *et al*., (2005)		XVE:	McGrath *et al*., (2005)	
AGI code	Gene name	JSI-1	MeJA	*A.Brassicicola*	JSI-1	MeJA	*A.Brassicicola*
AT5G61590	ERF107	2,05	-	-	5,64E-05	-	-
AT5G07580	ERF106	1,84	-	-	1,30E-05	-	-
AT5G61600	ERF104	2,81	-	-	9,49E-13	-	-
AT5G47230	ERF5	2,58	-	-	6,03E-17	-	-
AT4G17490	ERF6	3,38	-	3,39	2,67E-15	-	3,23E-02
AT4G17500	ERF-1	2,94	3,69	2,28	3,81E-23	1,59E-03	1,16E-02
AT5G47220	ERF2	2,84	3,53	2,17	2,82E-12	2,80E-04	2,05E-03
AT1G03800	ERF10	4,98	2,67	-	1,24E-08	1,12E-02	-
AT5G64750	ABR1	3,45	-	-	4,37E-19	-	-
AT5G61890	ERF114	17,50	-	-	2,25E-46	-	-
AT1G71520		8,43	-	-	9,16E-31	-	-
AT1G74930	ORA47	4,03	-	-	1,52E-13	-	-
AT4G06746[2]	RAP2.9	2,05	1,69	-	4,21E-04	3,88E-02	-
AT5G67190	DEAR2	2,04	-	-	6,67E-04	-	-
AT4G11280[2]	ACS6	2,42	-	-	2,13E-15	-	-
AT1G73500	MKK9	4,55	-	-	3,65E-62	-	-
AT1G18330	RVE7	2,00	-	-	7,81E-10	-	-
AT3G15356[2]	LEC	2,25	-	-	4,64E-16	-	-
AT5G05730[1]	ASA1	1,71	-	-	1,44E-03	-	-
AT3G50060	MYB77	2,61	-	-	1,26E-09	-	-
AT3G51430	SSL5	1,85	-	-	3,81E-04	-	-
AT5G17490	RGL3	2,30	-	-	3,73E-05	-	-
AT4G37260	MYB73	2,32	-	-	8,19E-05	-	-
AT1G71030	MYBL2	3,41	-	-	4,24E-07	-	-
AT1G15520	ABCG40	4,03	-	-	1,92E-11	-	-
AT1G01480	ACS2	2,93	-	-	3,50E-05	-	-
AT4G08040	ACS11	3,15	-	-	3,39E-07	-	-
AT4G11650[1,2]	OSM34	2,98	-	-	8,67E-05	-	-
AT4G37990[1]	ELI3-2	59,88	-	-	1,42E-31	-	-
AT1G67980[1]	CCOAMT	9,55	-	-	2,14E-12	-	-
AT1G75040[1]	PR5	3,85	-	-	4,64E-04	-	-
AT1G30720[1]		3,08	-	-	4,10E-17	-	-
AT5G20230[1]	BCB	5,85	-	-	1,56E-22	-	-
AT2G29460[1]	GSTU4	4,46	-	-	5,06E-21	-	-
AT2G02930[1,2]	GSTF3	4,81	-	-	1,46E-31	-	-
AT2G30770[1]	CYP71A13	17,74	-	-	7,83E-05	-	-
AT2G45570[1]	CYP76C2	7,53	-	-	3,82E-19	-	-
AT5G44420[1,2,3]	PDF1.2	5,69	-	-	2,38E-02	-	-
AT4G27410[1]	RD26	2,68	-	-	8,95E-15	-	-
AT4G37370[1]	CYP81D8	17,06	-	-	1,06E-233	-	-
AT5G07440[1]	GDH2	1,73	-	-	7,50E-06	-	-
AT3G48360[1]	BT2	1,92	-	-	3,50E-04	-	-

Continuation of **Table S3**

AGI code	Gene name	Fold change XVE: JSI-1	Fold change McGrath *et al*., (2005) MeJA	Fold change McGrath *et al*., (2005) *A. Brassicicola*	*P* value XVE: JSI-1	*P* value McGrath *et al*., (2005) MeJA	*P* value McGrath *et al*., (2005) *A. Brassicicola*
AT1G03220[1]		3,28	-	-	9,39E-95	-	-
AT5G26340[1]	MSS1	2,09	-	-	1,55E-06	-	-
AT2G14610[1]	PR1	226,45	-	-	7,82E-17	-	-
AT1G30730[1]		10,29	-	-	8,53E-52	-	-
AT3G09010[1]		2,51	-	-	1,44E-07	-	-
AT2G43570[1]	CHI	3,75	-	-	8,55E-08	-	-
AT1G30700[1]		2,31	-	-	1,02E-10	-	-
AT4G03420[1]		1,84	-	-	8,20E-05	-	-
AT1G78860[1]		1,96	-	-	1,77E-07	-	-
AT1G03230[1]		1,46	-	-	3,50E-26	-	-
AT5G25930[1]		2,36	-	-	9,42E-09	-	-
AT4G04610[1]	APR1	2,30	-	-	3,05E-10	-	-
AT4G25900[1]		1,92	-	-	1,85E-08	-	-
AT3G08760[1]	ATSIK	1,71	-	-	2,20E-04	-	-
AT3G16530[2]		4,15	-	-	5,79E-98	-	-
AT1G72910[2]		2,28	-	-	5,55E-05	-	-
AT5G03350[2]	LLP	11,15	-	-	5,18E-09	-	-
AT5G27420[2]	CNI1	2,86	-	-	2,03E-09	-	-
AT2G23770[2]	LYK4	2,28	-	-	5,88E-15	-	-
AT5G61160[2]	AACT1	3,49	-	-	1,31E-05	-	-
AT1G15125[2]		4,24	-	-	1,17E-35	-	-
AT1G66700[2]	PXMT1	3,36	-	-	1,80E-04	-	-
AT4G21990[2]	APR3	1,92	-	-	1,50E-03	-	-
AT1G67810[2]	SUFE2	2,16	-	-	9,06E-05	-	-
AT2G29350[1,2]	SAG13	48,14	-	-	2,01E-10	-	-
AT4G02520[2]	GSTF2	4,38	-	-	2,00E-29	-	-
AT1G69920[2]	GSTU12	10,12	-	-	2,66E-06	-	-
AT1G02930[2]	GSTF6	4,48	-	-	4,71E-20	-	-
AT1G02920[1,2]	GSTF7	5,89	-	-	8,17E-30	-	-
AT3G26200[2]	CYP71B22	2,70	-	-	3,18E-03	-	-
AT3G23550[2]	DTX18	2,62	-	-	2,77E-05	-	-
AT3G56710[2]	SIB1	6,77	-	-	4,23E-09	-	-
AT5G25250[2]	FLOT1	4,02	-	-	2,62E-24	-	-
AT1G26380[2]	FOX1	165,06	-	-	7,92E-124	-	-
AT5G42530[2]		18,85	-	-	1,98E-12	-	-

Genes upregulated in *A. thaliana* XVE-Jsi1-mCherry lines upon Jsi1 induction. [1]Genes upregulated in XVE-Jsi1-mCherry lines shared with *A. thaliana* 35S-ERF1 line (Lorenzo *et al*., 2003), and/or *A. thaliana* XVE-ORA59 line[2] (Pré *et al*., 2008). [3]PDF1.2 induction was not detected in the RNAseq of Jsi1 lines but was detected in the qRT-PCR assay. Fold change and *P* values of genes upregulated by methyl jasmonate (MeJa) and/or *Alternaria Brassicicola* (*A. Brassicicola*) were obtained from McGrath *et al*., (2005).

Table S4. Gene upregulated in *A. thaliana* XVE-jsi1mCh lines upon Jsi1 expression enriched for ERFs transcription binding sites.

Promoter Analysis[1]	AGI code
ERF3 ERF4 ERF8 ERF10 ERF11	AT5G43980
	AT3G57690
	AT5G62770
	AT1G12940
	AT4G39980
	AT2G36640
	AT1G11260
	AT1G71770
	AT4G06746
	AT1G22110
	AT1G16130
	AT2G33580
	AT4G17500
	AT4G24110
	AT5G52300
	AT1G52200
	AT4G39955
	AT1G30780
	AT4G24570
	AT2G29670
	AT5G67430
	AT1G80760
	AT3G44830
	AT1G12950
	AT2G30550
	AT1G69600
	AT3G29670
	AT1G03220
	AT3G55840
	AT2G42760
	AT5G67420
	AT5G67190
	AT5G39020
	AT5G50450
	AT5G16370
	AT5G09800
	AT2G38290
	AT1G75000
	AT3G10720

Continuation of **Table S4**

Promoter Analysis[1]	AGI code
ERF3 ERF4 ERF8 ERF10 ERF11	AT3G07350
	AT5G62280
	AT2G18700
	AT1G15840
	AT3G03440
	AT1G23760
	AT1G30730
	AT2G45760
	AT1G06570
	AT1G55850
	AT5G64720
	AT1G76600
	AT5G57220
	AT3G53040
	AT3G04640
	AT5G64750
	AT1G56060
	AT1G03800
	AT1G30720
	AT5G63160
	AT3G10040
	AT3G57240
	AT4G18170
	AT2G23320
	AT2G34355
	AT3G52480
	AT4G17490
	AT5G58770
	AT2G24550
	AT1G23390
	AT5G47220
	AT1G73880
	AT5G16340
	AT5G06320
	AT1G05300
	AT3G03470
	AT1G30740
	AT2G19500
	AT4G37260
	AT1G02660
	AT1G31290

Continuation of **Table S4**

Promoter Analysis[1]	AGI code
ERF3 ERF4 ERF8 ERF10 ERF11	AT2G41905
	AT4G00390
	AT1G71030
	AT1G78410
	AT1G75040
ERF3 ERF4 ERF8 ERF11	AT5G42380
	AT5G56270
	AT3G56620
	AT2G44578
	AT5G43590
	AT4G28420
	AT5G66070
	AT5G20250
	AT1G23550
	AT1G20310
	AT1G74790
	AT3G56880
	AT5G48560
	AT5G61600
ERF3 ERF4 ERF10 ERF11	AT3G56710
	AT1G49780
ERF4 ERF8 ERF10 ERF11	AT1G21680
	AT3G50060
	AT5G33290
	AT5G60680
	AT1G69920
	AT3G51190
	AT3G05200
	AT4G20830
	AT2G17880
	AT5G10760
	AT3G16530
	AT2G32150
	AT1G28480
	AT3G54140
	AT5G48430
	AT4G11650
	AT1G30280
	AT1G19380
	AT1G53080
	AT3G04050

Continuation of **Table S4**

Promoter Analysis[1]	AGI code
ERF4 ERF8 ERF10 ERF11	AT4G21320
	AT3G53960
	AT1G01480
	AT2G41410
	AT5G63130
	AT3G56400
	AT5G22140
	AT1G11210
	AT3G19010
	AT4G23810
	AT5G01330
	AT4G37690
	AT5G19120
	AT1G10140
	AT1G14890
	AT5G17490
	AT4G12330
	AT2G18620
ERF3 ERF4 ERF11	AT1G77120
	AT1G09500
ERF3 ERF8 ERF11	AT1G75960
ERF4 ERF8 ERF10	AT1G72100
	AT5G05440
ERF4 ERF8 ERF11	AT2G23150
	AT4G01870
	AT3G45680
	AT5G03350
	AT4G29610
	AT1G07160
	AT5G10625
	AT5G10380
	AT2G38640
	AT1G71140
	AT2G32680
	AT5G41750
	AT1G73500
	AT5G61890
	AT1G55910
	AT5G63790
	AT1G02450
	AT4G03460
	AT2G32800

Continuation of **Table S4**

Promoter Analysis[1]	AGI code
ERF4 ERF8 ERF11	AT4G32780
ERF4 ERF10 ERF11	AT1G14880
	AT4G30450
	AT2G25900
	AT3G09940
	AT5G17760
	AT1G17310
	AT1G14200
ERF8 ERF10 ERF11	AT2G47140
	AT1G22220
	AT1G03230
ERF3 ERF11	AT5G54300
ERF4 ERF8	AT1G23710
ERF4 ERF10	AT2G40000
ERF4 ERF11	AT1G25550
	AT1G56250
	AT1G72900
	AT5G25240
	AT3G62950
	AT5G05730
	AT3G53160
	AT3G46090
	AT2G34870
	AT1G74940
	AT1G04770
	AT2G33160
	AT2G44370
	AT1G33475
	AT3G20395
	AT3G02800
ERF8 ERF10	AT4G28350
	AT4G29190
	AT4G26080
	AT1G21670
	AT4G28240
	AT5G05340
ERF8 ERF11	AT4G14680
	AT5G26340
	AT3G48850
	AT1G08050
	AT4G04510
	AT3G43850

Continuation of **Table S4**

Promoter analysis[1]	AGI code
ERF10 ERF11	AT1G70920
	AT2G43570
ERF3	AT1G65730
ERF4	AT2G18540
	AT3G11840
ERF8	AT4G27410
	AT4G36040
	AT5G07550
	AT5G60900
	AT2G19810
	AT2G34010
	AT3G50770
	AT2G38820
	AT5G47230
	AT4G21380
	AT3G45660
	AT1G04180
	AT1G15010
	AT1G35350
	AT1G62940
	AT1G16150
	AT2G03170
	AT1G35140
	AT3G02040
	AT5G38490
	AT2G27830
	AT2G20670
	AT3G59080
ERF10	AT3G20300
	AT4G22530
	AT2G46150
	AT4G04500
	AT1G69150
	AT5G49620
	AT4G23200
	AT5G53870
	AT3G18250
	AT5G04340
	AT3G17520
	AT1G30700
	AT4G10960
	AT5G10830

Continuation of **Table S4**

Promoter Analysis[1]	AGI code
ERF10	AT1G24130
	AT1G71520
	AT4G39830
	AT5G41610
	AT4G39610
	AT2G41100
ERF11	AT3G63380
	AT3G15670
	AT1G33960
	AT4G11280
	AT3G13840
	AT3G26830
	AT3G10985
	AT5G44567
	AT1G22890
	AT5G63800
	AT4G03420
	AT5G47850
	AT5G21940
	AT1G49640
	AT3G61390
	AT1G25400

[1]Direct target genes of ERFs were obtained from published DAP-seq data (O'Malley *et al.*, 2016).

References

Aichinger C, Hansson K, Eichhorn H, Lessing F, Mannhaupt G, Mewes W, Kahmann R. 2003. Identification of plant-regulated genes in *Ustilago maydis* by enhancer-trapping mutagenesis. *Molecular Genetics and Genomics* **270**: 303-314.

Ausubel, F.M., Brenz, R., Kongston, R.E., Moore, D.D., Seidmann, J.G., Smith, J.A. & K. Strukl. 1987. Current protocols in molecular microbiology. John Wiley & Sons, Inc., USA

Bouton C, King RC, Chen H, Azhakanandam K, Bieri S, Hammond-Kosack KE, Kanyuka K. 2018. Foxtail mosaic virus: A viral vector for protein expression in cereals. *Plant physiology* **177**: 1352-1367.

Brown RL, Kazan K, McGrath KC, Maclean DJ, Manners JM. 2003. A role for the GCC-box in jasmonate-mediated activation of the *PDF1. 2* gene of Arabidopsis. *Plant physiology* **132**, 1020-1032.

Djamei A, Schipper K, Rabe F, Ghosh A, Vincon V, Kahnt J, Osorio S, Tohge T, Fernie AR, Feussner I et al. 2011. Metabolic priming by a secreted fungal effector. *Nature* **478**: 395-398.

Fernández-Bautista N, Domínguez-Núñez JA, Moreno MC, Berrocal-Lobo M. 2016. Plant tissue trypan blue staining during phytopathogen infection. *The plant journal* **24**: 1-7.

Iven T, König S, Singh S, Braus-Stromeyer SA, Bischoff M, Tietze LF, Braus GH, Lipka V, Feussner I, Dröge-Laser W. 2012. Transcriptional activation and production of tryptophan-derived secondary metabolites in Arabidopsis roots contributes to the defense against the fungal vascular pathogen *Verticillium longisporum*. *Molecular Plant* **5**: 1389-1402.

Kämper J, Kahmann R, Bölker M, Ma LJ, Brefort T, Saville BJ, Banuett F, Kronstad JW, Gold SE, Müller O *et al*. 2006. Insights from the genome of the biotrophic fungal plant pathogen *Ustilago maydis*. *Nature* **444**: 97-101.

Kim JH, Lee S-R, Li L-H, Park H-J, Park J-H, Lee KY, Kim M-K, Shin BA, Choi S-Y. 2011. High Cleavage Efficiency of a 2A Peptide Derived from Porcine Teschovirus-1 in Human Cell Lines, Zebrafish and Mice. *PLOS ONE* **6**: e18556.

Lanver D, Müller AN, Happel P, Schweizer G, Haas FB, Franitza M, Pellegrin C, Reissmann S, Altmüller J, Rensing SA *et al*. 2018. The biotrophic development of *Ustilago maydis* studied by RNA-Seq analysis. *The Plant Cell* **30**: 300-323.

Lampropoulos A, Sutikovic Z, Wenzl C, Maegele I, Lohmann JU, Forner J. 2013. GreenGate-A novel, versatile, and efficient cloning system for plant transgenesis. *PLoS One* **8**:e83043.

Liu X, Sun Y, Kørner CJ, Du X, Vollmer ME, Pajerowska-Mukhtar KM. 2015. Bacterial Leaf Infiltration Assay for Fine Characterization of Plant Defense Responses using the *Arabidopsis thaliana-Pseudomonas syringae* Pathosystem. *JoVE* e53364.

Lin F, Jiang L, Liu Y, Lv Y, Dai H, Zhao H. 2014. Genome-wide identification of housekeeping genes in maize. *Plant molecular biology* **86**: 543-554.

Lorenzo O, Piqueras R, Sánchez-Serrano JJ, Solano R. 2003. ETHYLENE RESPONSE FACTOR1 integrates signals from ethylene and jasmonate pathways in plant defense. *The Plant Cell* **15**: 165-178.

McGrath KC, Dombrecht B, Manners JM, Schenk PM, Edgar CI, Maclean DJ, Scheible WR, Udvardi MK, Kazan K. 2005. Repressor-and activator-type ethylene response factors functioning in jasmonate signaling and disease resistance identified via a genome-wide screen of Arabidopsis transcription factor gene expression. *Plant physiology* **139**: 949-959.

O'Malley RC, Huang SSC, Song L, Lewsey MG, Bartlett A, Nery JR, Galli M, Gallavotti A, Ecker JR. 2016. Cistrome and epicistrome features shape the regulatory DNA landscape. *Cell* **165**: 1280-1292.

Pré M, Atallah M, Champion A, De Vos M, Pieterse CM, Memelink J. 2008. The AP2/ERF domain transcription factor ORA59 integrates jasmonic acid and ethylene signals in plant defense. *Plant physiology* **147**: 1347-1357.

Sambrook J, Russell DW. 2006. Purification of Nucleic Acids by Extraction with Phenol:Chloroform. *Cold Spring Harbor Protocols* 2006: pdb.prot4455.

Schellingen K, Van Der Straeten D, Vandenbussche F, Prinsen E, Remans T, Vangronsveld J, Cuypers A. 2014. Cadmium-induced ethylene production and responses in Arabidopsis thaliana rely on ACS2 and ACS6 gene expression. *BMC Plant Biology* **14.1**: 214.

Uhse S, Pflug FG, Stirnberg A, Ehrlinger K, von Haeseler A, Djamei A. 2018. In vivo insertion pool sequencing identifies virulence factors in a complex fungal–host interaction. *PLoS biology* **16**: e2005129.

Zhang T, Maruhnich SA, Folta KM. 2011. Green light induces shade avoidance symptoms. *Plant Physiology* **157**: 1528-1536.

Zienkiewicz A, Gömann J, König S, Herrfurth C, Liu Y-T, Meldau D, Feussner I. 2020. Disruption of Arabidopsis neutral ceramidases 1 and 2 results in specific sphingolipid imbalances triggering different phytohormone-dependent plant cell death programs. *New Phytologist* **226**: 170-188.
